# Thermal stabilization of the deglycating enzyme Amadoriase I by rational design

**DOI:** 10.1038/s41598-018-19991-x

**Published:** 2018-02-14

**Authors:** Federica Rigoldi, Stefano Donini, Francesca Giacomina, Federico Sorana, Alberto Redaelli, Tiziano Bandiera, Emilio Parisini, Alfonso Gautieri

**Affiliations:** 1 Biomolecular Engineering Lab, Dipartimento di Elettronica, Informazione e Bioingegneria, Politecnico di Milano, Piazza Leonardo da Vinci 32, 20133 Milano, Italy; 2 Center for Nano Science and Technology @Polimi, Istituto Italiano di Tecnologia, Via G. Pascoli 70/3, 20133 Milano, Italy; 3 D3-PharmaChemistry, Istituto Italiano di Tecnologia, Via Morego 30, 16163 Genova, Italy

## Abstract

Amadoriases are a class of FAD-dependent enzymes that are found in fungi, yeast and bacteria and that are able to hydrolyze glycated amino acids, cleaving the sugar moiety from the amino acidic portion. So far, engineered Amadoriases have mostly found practical application in the measurement of the concentration of glycated albumin in blood samples. However, these engineered forms of Amadoriases show relatively low absolute activity and stability levels, which affect their conditions of use. Therefore, enzyme stabilization is desirable prior to function-altering molecular engineering. In this work, we describe a rational design strategy based on a computational screening method to evaluate a library of potentially stabilizing disulfide bonds. Our approach allowed the identification of two thermostable Amadoriase I mutants (SS03 and SS17) featuring a significantly higher *T*_50_ (55.3 °C and 60.6 °C, respectively) compared to the wild-type enzyme (52.4 °C). Moreover, SS17 shows clear hyperstabilization, with residual activity up to 95 °C, whereas the wild-type enzyme is fully inactive at 55 °C. Our computational screening method can therefore be considered as a promising approach to expedite the design of thermostable enzymes.

## Introduction

**Figure 1:**
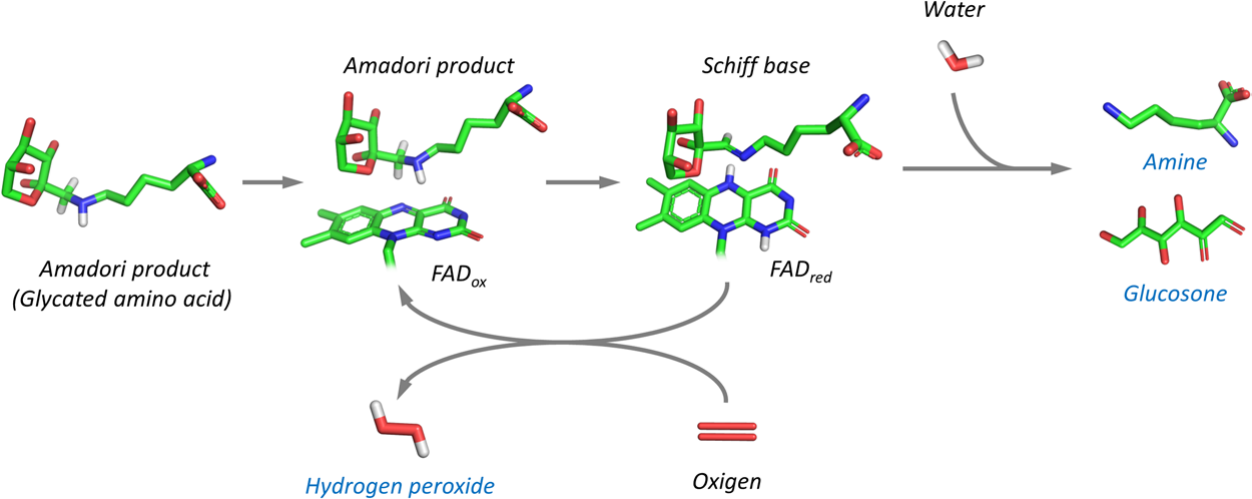
Schematic of the amino acid deglycation reaction catalyzed by Amadoriase I. The enzyme catalyzes the oxidation of the C-N bond between the nitrogen of the amino acid moiety of the Amadori product and C1 of the fructosyl portion. The reaction results in the Schiff base which is hydrolyzed to yield glucosone and a free amino acid. The reduced FAD is oxidized by an oxygen molecule with the release of hydrogen peroxide. Only relevant hydrogen atoms are shown. Blue font highlights the products of the enzymatic catalysis.

Amadoriases, also known as Fructosyl Amino Acid Oxidases (FAOX)^1–3^, are a class of enzymes that are found in fungi and bacteria and that are able to cleave low molecular weight Amadori product (i.e., glycated amino acids) to yield a free amine, glucosone and hydrogen peroxide^4,5^ (Fig. 1).

Today, members of this class of enzymes, commonly referred to as Fructosyl Peptide Oxidases (FPOX)^6–8^, are used in the detection of glycated haemoglobin (HbA1c), a long term marker for diabetes (the half-life of HbA1c is 3 months). Such diabetes monitoring kits are based on a first proteolytic digestion of HbA1c, which releases single amino acids from the protein, including its N-terminal glycation-prone valine. Then, the FPOX enzyme binds glycated valine and hydrolyzes it producing hydrogen peroxide which, in turn, is measured in a colorimetric assay using horseradish peroxidase and a suitable chromophore^9^. Using a similar mechanism, the Amadoriase I enzyme is of potential use in the detection of glycated albumin, a short to mid-term glycemic marker for diabetes (glycated albumin has a 3 weeks half-like)^10^. However, a common problem with biosensors is the long-term stability of their biological components. Hence, a stabilized Amadoriase enzyme will improve biosensor stability during transport and storage and the overall shelf-life.

Interestingly, Amadoriase enzymes are also regarded as promising therapeutic tools for the prevention or the reduction of protein glycation in biological tissues^11^. Glycation is the spontaneous, non-enzymatic and irreversible reaction between a sugar moiety and a protein, leading to a covalent adduct^12^. By modifying the chemistry of functional proteins^13,14^, glycation leads to a cascade of adverse clinical outcomes including arterial stiffening^15^, atherosclerosis^16^, nephropathy^17^, retinopathy^18^ and neuropathy^19^. So far, the use of Amadoriases to prevent protein glycation is precluded because these enzymes show no significant activity on intact proteins^20,21^ owing to their buried active site location and to the narrow tunnel that provides access to the catalytic pocket^18,19^. A stabilized form of Amadoriase may therefore help to set the stage for a further, more extensive engineering approach to the design of protein deglycation tools.

A further potential application of thermostable Amadoriases concerns the prevention of acrylamide formation in processed food. Thermal treatments used in food manufacturing (such as baking, toasting, frying, and roasting) boost the Maillard reaction between reducing sugars and amino acids, which gives aromas and taste. However, the reaction between sugars and asparagine amino acids yields acrylamide, a carcinogenic compound^22,23^. This outcome is particularly relevant in fried potatoes, bakery products and coffee, and currently there are no viable strategies to mitigate acrylamide formation while preserving the desired properties of processed food^24,25^. A thermostable Amadoriase enzyme is a potential tool to be used in food processing to limit the Maillard reaction on single amino acids and thus acrylamide formation.

All the aforementioned applications of Amadoriases can benefit from or necessitate the stabilization of the enzymes. In this study, we apply a novel rational design approach to the stabilization of wild-type Amadoriase I from *Aspergillus fumigatus*. In general, any modification of the properties of a protein can in principle be achieved through the use of different rational approaches. Indeed, several computational methods to predict protein stability or changes in stability upon natural sequence mutation have been developed and tested^26,27^. However, the performance of these popular methods is still relatively weak^28^. Usually, the rational design of proteins with improved stability involves optimization of charge-charge interactions, saturation mutagenesis of residues with high crystallographic B-factors^29^, methods based on protein simulation and calculation of free energies^30^, phylogenetic analysis^31^, comparison to homologous proteins including the ultra-stable proteins of thermophilic organisms^32^, or structure-based computational design^33,34^. As expected, all these methods have a relatively high probability of inadvertently introducing disruptive mutations^33,35^. Hence, efforts to stabilize large proteins have also been based on library approaches aimed at identifying optimal combinations of stabilizing mutations^36–38^. However, such approaches are laborious and impractical for proteins without established medium-to-high-throughput screening protocols.

In an effort to address this issue efficiently, we developed a high-throughput computational screening method based on Molecular Dynamics (MD) simulations whereby the evaluation of a library of potentially stabilizing disulfide bonds (obtained by SSBOND software^39^) allows for the selection of a few candidates to be produced and tested experimentally. Here, we report on the identification, production and enzymatic characterization of four Amadoriase I mutants, two of which show a remarkable increase in thermal stability compared to the wild type enzyme.

## Results

### Computational design and screening of disulfide bonds

**Figure 2:**
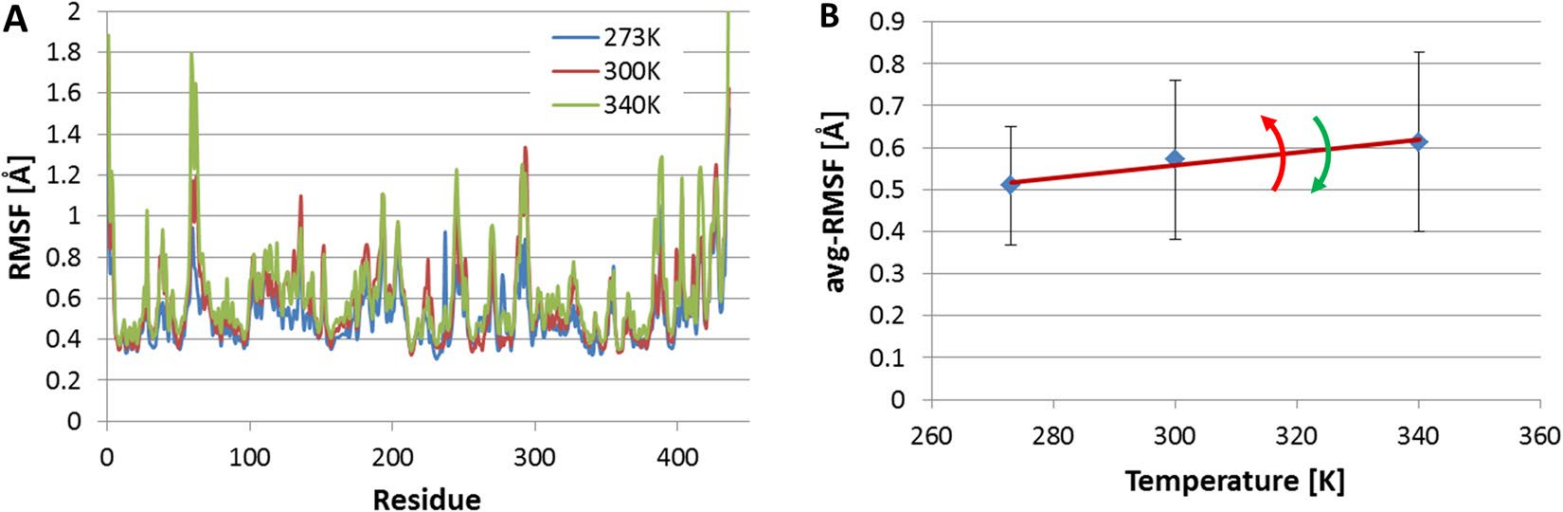
MD-based screening of mutants. (**A**) RMSF of WT-Amadoriase I at different temperatures, showing how higher temperatures induce an increase in the baseline but also a steep increase in specific regions of the protein (e.g., residue from 60 to 70). (**B**) avg-RMSF of WT-Amadoriase I at increasing temperatures. Here, the slope is considered as a proxy of the sensitivity to thermal stress and it is used to filter out mutations that badly affect thermal stability. In particular, enzyme mutants showing larger slopes are considered less stable than the WT, whereas enzyme mutants showing smaller slopes are considered as promising thermostable variants and thus experimentally tested.

Using the software SSBOND^39,40^ we obtained a list of 19 possible disulfide bonds sites. Starting from the crystal structure of Amadoriase I (PDB id: 4WCT)^41^, we built the molecular models of the wild-type (WT) and those of the 19 Amadoriase I variants (SS01 to SS19), each featuring a different disulfide bond, and we screened them using MD simulations. The root mean square fluctuation (RMSF) was calculated for three different temperatures (270, 300 and 340 K) (Fig. 2 Panel A). The average RMSF (avg-RMSF) as a function of the temperature (Fig. 2 Panel B) was then interpolated with a linear equation. The slope (*λ*) represents the index that we used to discriminate the stabilized mutants. SS-variants with a larger *λ* with respect to WT were discarded while those with *λ* lower than that of the WT were selected for experimental production.

**Figure 3:**
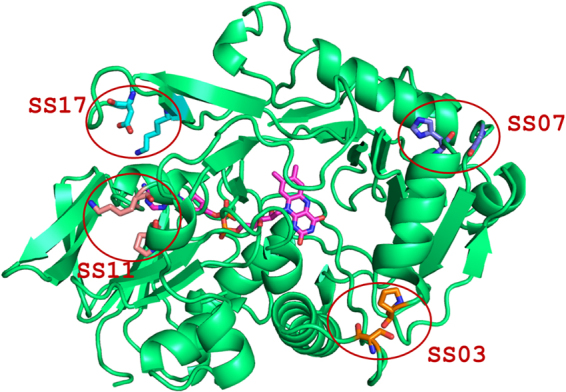
Amadoriase I enzyme and selected mutations. The crystal structure of WT Amadoriase I (PDB code: 4WCT) is shown in cartoon representation, while the residues that were mutated to Cys in the selected SS-variants are represented in sticks (for SS03 residues S67 and P121, for SS07 residues H106 and G150, for SS11 residues K233 and P404, for SS17 residues D295 and K303). The FAD cofactor is shown in purple sticks.

As shown in Table 1, 13 out of the 19 possible disulfide bonds were excluded on the basis of a *λ* value larger than *λ*_*WT*_. One variant (SS10) was excluded due to the poor fitting of avg-RMSF vs. temperature. We also excluded SS08 because the disulfide involved only residues from the rather floppy C-terminus and thus unlikely to stabilize the whole structure. At the end of our MD-based screening, four mutants (SS03, SS07, SS11 and SS17) were selected for experimental characterization (Fig. 3). The MD-based evaluation of each enzyme variant took approximately 10 h on a NVIDIA Tesla K80. We note that MD simulations of few nanoseconds are enough to capture local conformational changes and small loop rearrangements, while larger domain relaxation would require millisecond-long simulations, which are beyond the current state-of-the-art.

**Table 1:** Screening of SS-variants.

Enzyme	Mutated residues	λ	R^2^	p-value
*WT*	*—*	*0.00151*	*0.953*	
SS01*	S10, H216	0.00162	0.965	0.0407
SS02*	A29, L179	0.00201	0.996	0.0013
SS03	S67, P121	0.00151	0.995	0.8234
SS04*	A74, I399	0.00242	0.997	0.0004
SS05	Y95, A168	0.00187	0.993	0.8326
SS06*	G100, P260	0.00202	0.960	0.0028
SS07*	H106, G150	0.00115	0.997	0.0022
SS08*	K438, E441	0.00138	0.984	0.0187
SS09*	F148, W151	0.00181	0.972	0.0056
SS10*	V202, L263	0.00063	0.426	0.0021
SS11*	K233, P404	0.00134	0.924	0.0202
SS12*	K234, A407	0.00183	0.999	0.0027
SS13*	Q247, S337	0.00173	0.990	0.0070
SS14*	M248, V253	0.00202	0.939	0.0036
SS15*	C290, S304	0.00221	0.926	0.0026
SS16*	G432, Q435	0.00166	0.888	0.0475
SS17*	D295, K303	0.00118	0.993	0.0027
SS18*	R298, Q301	0.00187	0.999	0.0022
SS19*	L324, A332	0.00170	0.997	0.0080

As a result of the comparison of the λ value for the 19 SS-variants with λ_WT_, four mutants (SS03, SS07, SS08, SS17) were selected for experimental production and characterization. Statistically significant differences between the λ value of SS-variant and λ of WT are marked with (*).

### Production, purification and biochemical characterization of the Amadoriase variants

Based on our computational design and screening method, we proceeded with the production and characterization of the four most promising thermostable mutants (SS03, SS07, SS11, and SS17). In addition, we produced and characterized a mutant species (SSDM) featuring the combination of the two most effective disulfide bonds resulting from the first round of experimental characterization (i.e., SS03 and SS17).

**Figure 4:**
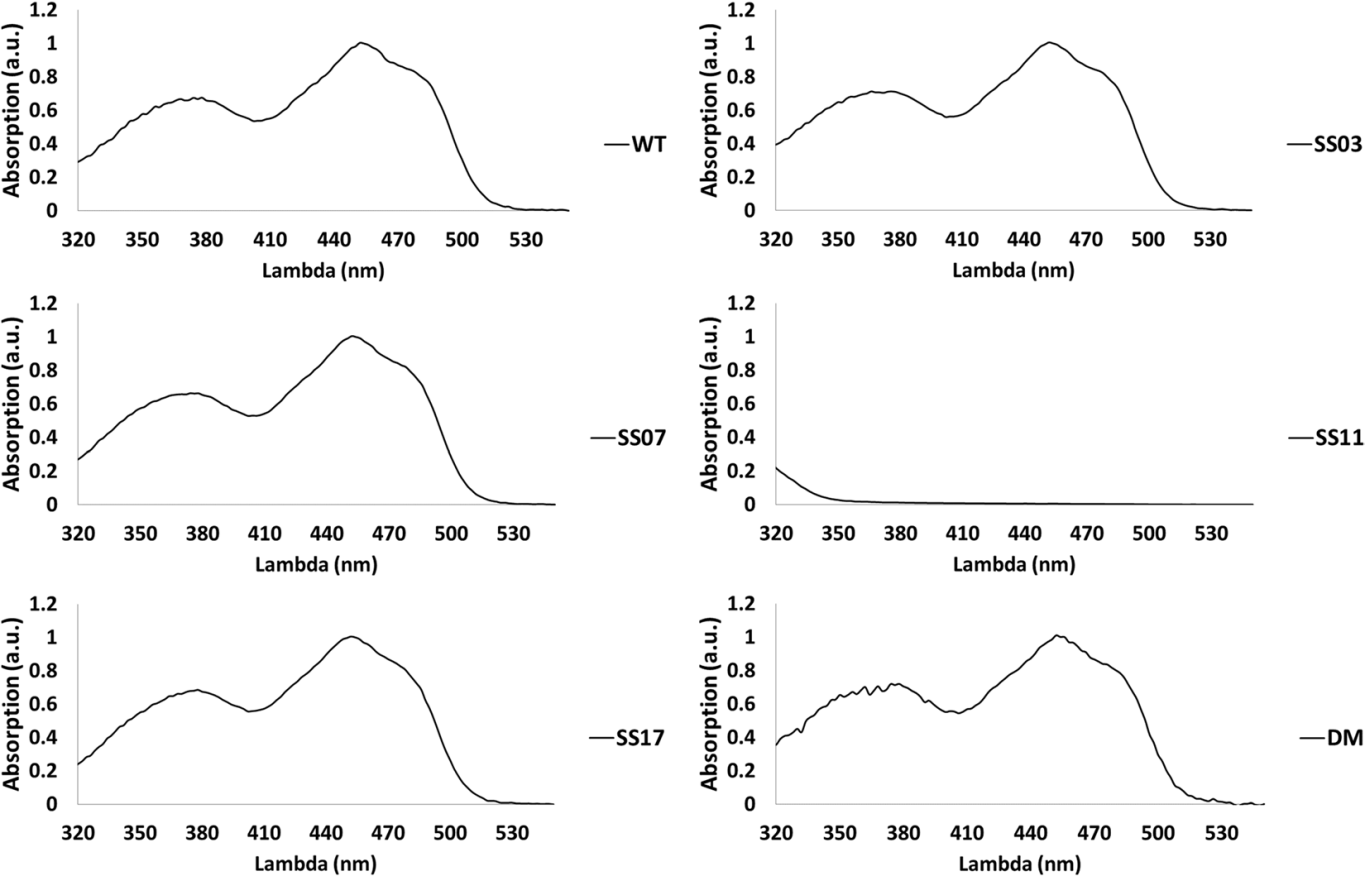
UV-vis absorption spectra. The UV-visible absorption spectra of WT and SS03, SS07, SS17, and SS19 variants show two prominent peaks (at 368 nm and 454 nm) typical of bound FAD cofactor. Conversely, the SS11 does not show the two relevant regions of the UV-visible spectrum, indicating that it does not bind the flavin cofactor.

We obtained pure and active Amadoriase I SS03, SS07, SS17, and SSDM mutants with an average yield of 30 mg per liter of bacterial culture. In contrast, the SS11 variant showed much lower expression levels (less than 1 mg/L) and no detectable activity. The four highly expressed proteins exhibit a bright yellow color. Their absorption spectrum, which features two major absorbance peaks, one at 368 and one at 454 nm (Fig. 4), indicates the presence of flavin as prosthetic group. Hence, the absence of these signature peaks in the absorption spectrum of the SS11 variant suggests no binding of FAD moiety by the protein. This feature, combined with the dramatically reduced amount of soluble protein expressed and the complete enzyme inactivation, suggests that the cysteine residues introduced in position 233 and 404 highly destabilize the enzyme. For these reasons, we discarded the SS11 mutant and we did not proceed any further with its characterization.

Apparent steady-state parameters (*K*_*m*_, k_*cat*_ and k_*cat*_/*K*_*m*_) calculated for all the mutants towards fructosyl-lysine were consistent with those reported in the literature for wild-type Amadoriases^9,20,42^ (Table 2). For the SS03 and the SS17 species, the mutations did not significantly affect the apparent steady state parameters relative to the WT. On the other hand, the SS07 mutant exhibited a two-fold increase in *K*_*m*_ as well as a four-fold decrease in catalytic efficiency compared to the WT. This may be due to a modification of the access tunnel to the catalytic pocket of the enzyme as a result of the introduction of a disulfide bond between residues H106 and G150, which are in close proximity to the entrance of the tunnel.

**Table 2:** Comparison of the apparent steady state parameters for wild type Amadoriase I to the mutated enzymes using fructosyl-lysine as substrate.

Enzyme	*K*_*m*_ [mM]	*k*_cat_ [s^−1^]	*k*_cat_/*K*_m_ [s^−1^ mM^−1^]
WT	0.51 ± 0.19	21.55 ± 3.08	41.68 ± 16.48
SS03	0.34 ± 0.13	21.90 ± 2.73	64.17 ± 25.5
SS07	0.99 ± 0.22	11.97 ± 1.69	12.02 ± 2.86
SS17	0.68 ± 0.18	22.91 ± 2.12	33.34 ± 10.16
SSDM	0.97 ± 0.22	14.50 ± 1.67	14.85 ± 3.85

Experiments were performed at 25 °C, as described in the material and methods section.

To support this hypothesis, we compared substrate tunnel dimension between the wild type enzyme and the SS07 mutant using CAVER 3.0.2 software^43^. For the analysis of the geometry of the tunnel, we removed the ions and water molecules that were included in the MD runs. In particular, we applied the same protocol developed in one of our previously published works^44^, which allowed us to identify the tunnel as a mean of the calculated tunnels in each frame of the MD trajectory. As a starting point for tunnel calculation we used the coordinate of the N5 atom of flavin, the enzyme’s cofactor. As a probe, we defined a sphere of 1.4 nm radius, a value comparable to that of the cyclic form of glucose. To describe the surface of the protein correctly, we used a shell depth of 6 Å and a shell radius of 4 Å. We set the tunnel clustering threshold to 5 Å. Tunnel calculations were done at 5 ns of the MD simulations at 300 K. Default values were used for all the other parameters. For comparison, Table 3 reports the dimensions of the substrate tunnel bottleneck of the wild type enzyme and the SS07 mutant.

**Table 3:** Comparison of the size of the substrate tunnel bottleneck in the wildtype enzyme and in the SS07 mutant.

Enzyme	*Average bottleneck [Å]*
WT	1.83 ± 0.17
SS07	1.57 ± 0.20

Our results show that MD simulations predict a local structural modification between two enzymes in the region of the substrate tunnel (see Fig. S1 in Supplementary Information). We suppose that this change could affect both the catalytic efficiency (since it involves the ligand pathway to the active pocket) and the thermal stability of the enzyme.

It is worth noting that, similarly to the SS07 mutant, also SSDM shows decreased catalytic efficiency and substrate affinity, suggesting that either the presence of both disulfide bonds results in some local structural perturbation that affects enzymatic activity or that no actual double SS bond formation occurs and the enzyme becomes partially destabilized by the introduced mutations.

**Figure 5:**
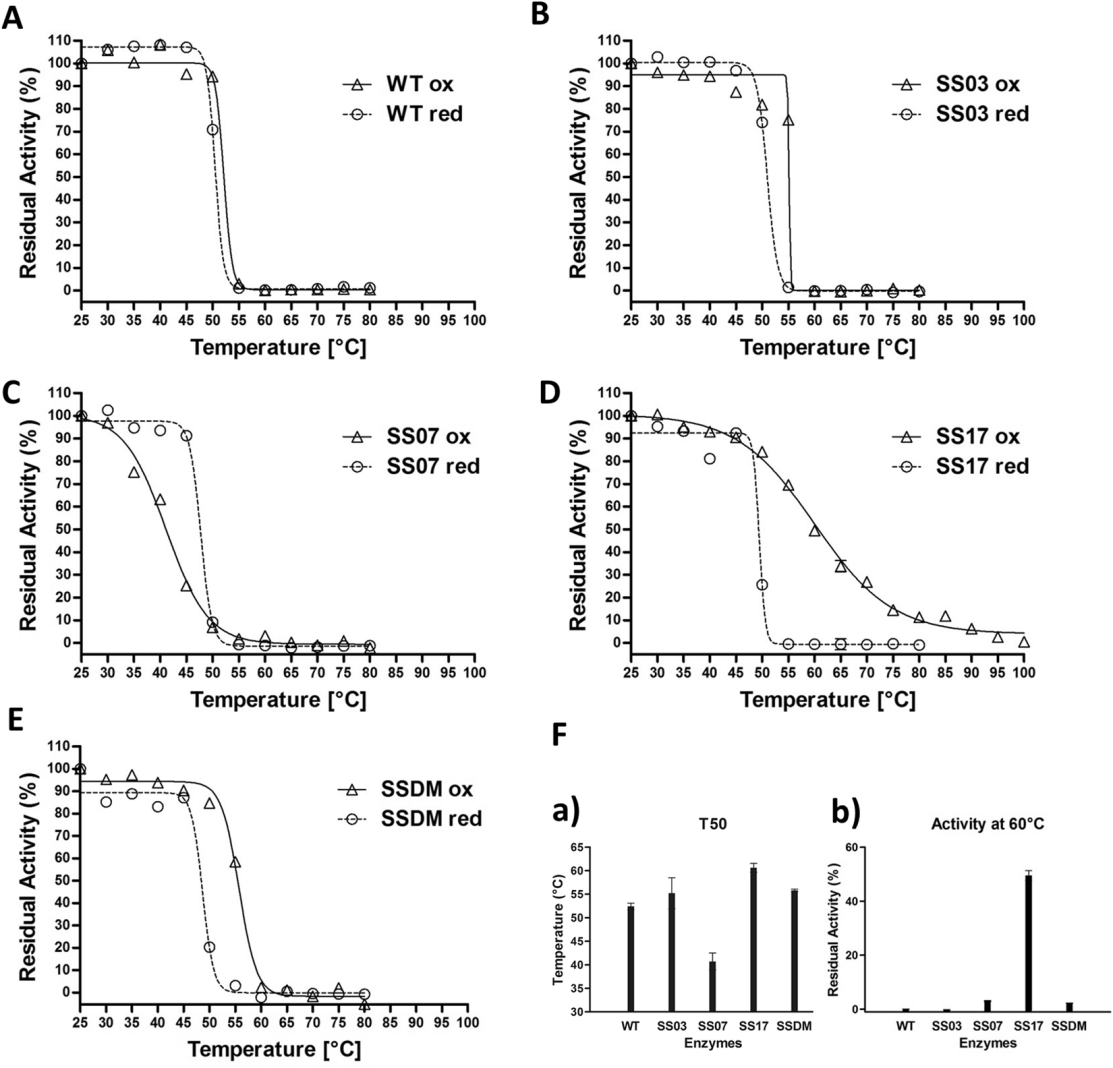
Temperature effect on the activity of the WT Amadoriase I and the mutants. (**A**–**E**) Residual activity of the oxidized form (triangle) and the reduced form (circle) as a function of the temperature for the WT enzyme and the tested SS-mutants. Enzymes were treated at temperatures ranging from 25 °C to 100 °C for 10 min before the activity assay. The residual activity at 25 °C, for each enzyme, is assumed as 100%. (**F**) Panel (a) Temperature at which each enzyme loses half of its initial activity (measured at 25 °C) Panel (b) Residual activity at 60 °C. The reported data are the mean values of three replicates ±SD (standard deviation).

The temperature effect on the WT and the mutants’ activity is shown in Fig. 5. For all enzyme variants we report the *T*_50_, i.e. the temperature at which the enzymes lose 50% of the activity with respect to the activity at 25 °C (Table 4). With the exception of the SS07 mutant, which shows a decreased stability, mutants SS03 and SS17 display a significant increase in *T*_50_ compared to the WT enzyme (3 °C and 8 °C, respectively). Interestingly, while all the enzymes, including the WT, lose completely their activity at temperatures ≥60 °C, the SS17 mutant retains a 50% residual activity at 60 °C and shows residual activity up to 95 °C. To confirm disulfide bond formation, we performed the same experiments also in the presence of 100 mM 1,4-dithiothreitol (DTT). Indeed, the reduced forms of all the SS-mutants lose their improved thermal resistance and behave very similarly to the WT. On the other hand, the SS07 is more stable in a reducing environment, further confirming the detrimental effect of the introduced disulfide bond in this species. Interestingly, the SSDM, which potentially carries both disulfide bonds of SS03 and SS17 does not display any cooperative effect relatively to the two single mutants; in fact, the measured *T*_50_ is consistent with the one obtained for the SS03 mutant, suggesting that only the C67-C121 disulfide bond may be actually formed.

**Table 4:** Absolute T_50_ and difference with respect to WT Amadoriase (ΔT_50_).

Enzyme	*T*_50_ [°C]	*ΔT*_50_ [°C]
WT	52.40 ± 0.69	—
SS03	55.25 ± 3.28	+2.85
SS07	40.76 ± 1.80	−11.64
SS17	60.62 ± 0.95	+8.22
SSDM	55.79 ± 0.25	+3.39

The reported data are the mean values of three replicates ±SD (standard deviation).

**Figure 6:**
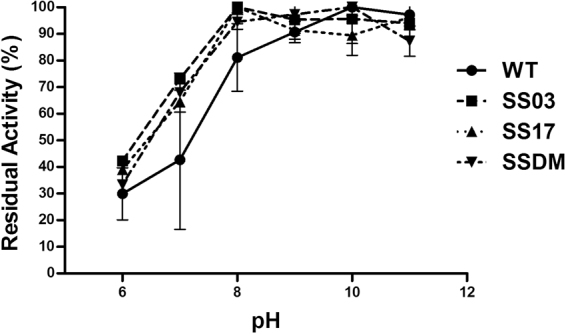
Effect of pH on the activity of the WT Amadoriase I and the thermally stabilized mutants. Activity was measured at 25 °C in a pH range from 6 to 11. Experimental conditions are described in the materials and methods section. The activity is normalized to the maximum activity for each enzyme. The reported data are the mean values of three replicates **±**SD (standard deviation).

The pH-activity profiles for the WT and all the thermally stabilized enzymes described herein (SS03, SS17, and SSDM) feature a rather similar behavior, showing a decrease in activity below pH 8.0 and a plateau above this value (Fig. 6). Therefore, the increase in thermal stability due to the introduction of disulfide bonds is not paralleled by any increase in resistance to a wider range of pH values compared to the WT.

### Crystal structures

**Figure 7:**
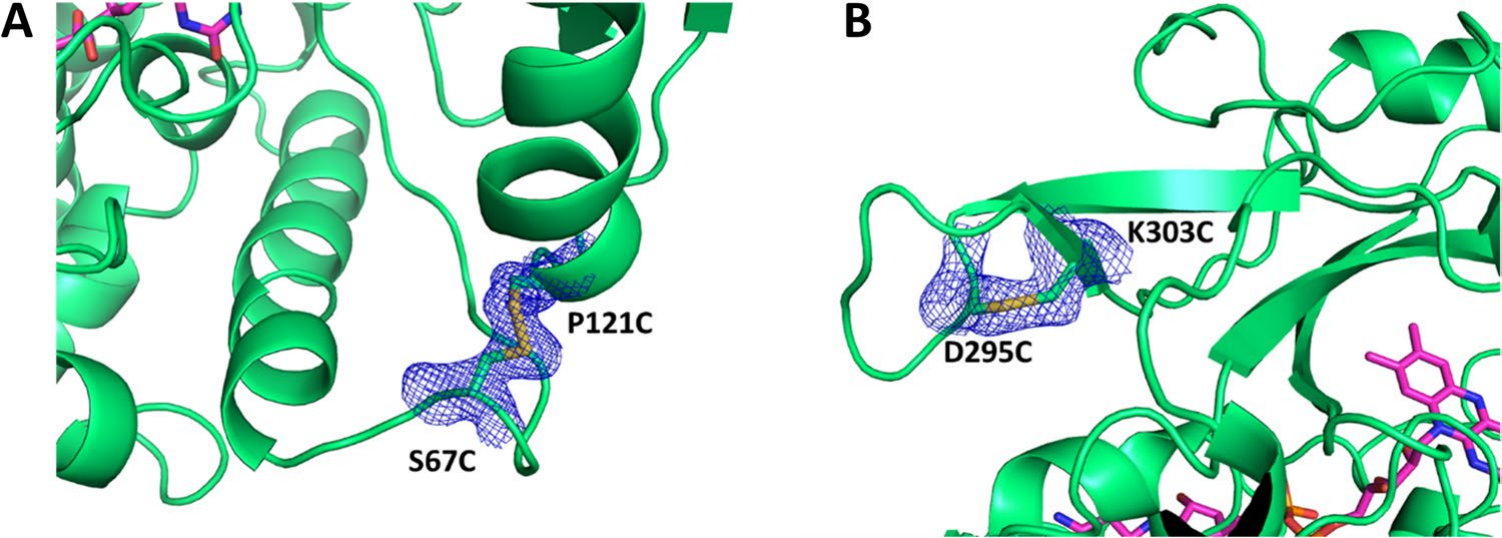
Detailed view of the electron density map in the region of the double cysteine mutation for the SS03 enzyme variant (Panel A) and for the SS17 enzyme variant (Panel B). Both maps show the clear formation of the disulfide bonds.

We used X-ray crystallography to confirm the introduction of the designed disulfide bonds in the mutant enzymes. The crystal structures of the SS03 and the SS17 mutants were determined at 2.15 and 2.85 Å resolution, respectively. Both structures feature two independent molecules in the asymmetric unit. However, while the SS03 mutant crystallizes in the same space group (orthorhombic P2_1_2_1_2_1_) as the wild type enzyme (PDB code: 4WCT), the SS17 variant shows a different packing arrangement as it crystallizes in the monoclinic space group P2_1_ (Fig. S2 of Supplementary Information). The disulfide bonds as they appear in the electron density maps of our two mutants are shown in Fig. 7.

The overall fold of the two mutants is very similar to that of the wild-type enzyme, with an RMSD for main chain atoms of 0.268 and 0.804 Å for SS03 and SS17, respectively.

**Figure 8:**
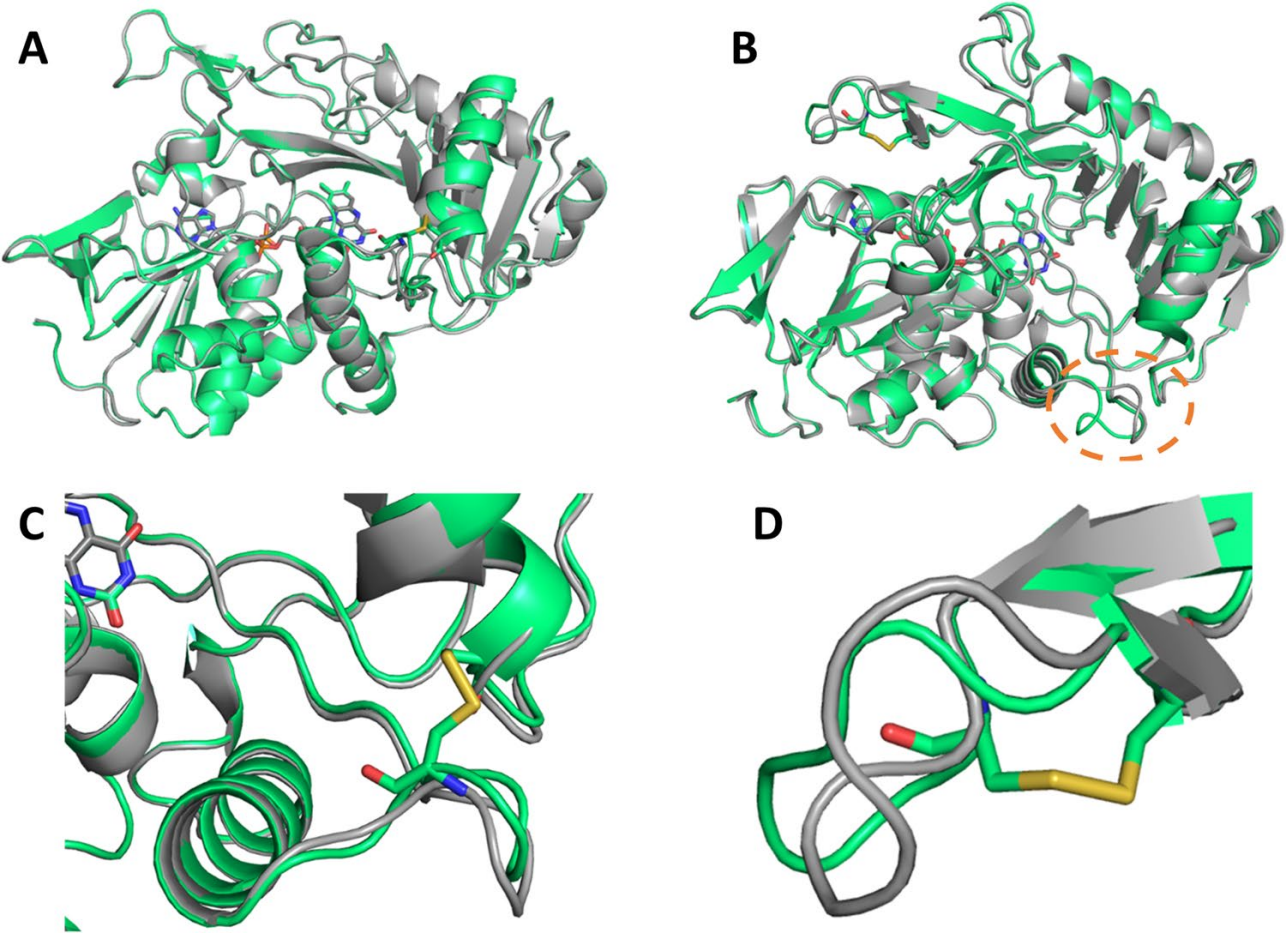
Superposition of WT enzyme (gray) with the disulfide mutants (green). The SS03 mutant (Panel A) presents no significant alteration of any part of the enzyme due to the introduction of the disulfide bond, including the region near the double cysteine mutation (Panel C). The SS17 mutant (Panel B) presents no significant alteration in the overall structure of the enzyme and in the catalytic pocket. However, it shows a structural rearrangement close to the introduced mutations (Panel D) and a significant displacement of a loop (residues 63 to 69) that is far from the introduced mutation (orange circle in Panel B).

No significant variation in the geometry of the catalytic pocket can be observed between the two mutants and the wild type enzyme, demonstrating that indeed the introduction of these disulfide bonds, which is intended for thermal stabilization only, does not alter the architecture of the active site of the enzyme (Fig. 8, Panel A and B). This observed structural invariability in the catalytic pocket correlates with the activity profile of the mutant enzymes, which are by and large similar to the wild type species up to the temperature at which the natural Amadoriase I maintains its fold and activity.

To assess the results of the MD simulations in terms of structural conformation, we calculated the Root Mean Square Deviation (RMSD) for main-chain atoms between the crystallographic structures and the final conformation obtained by MD at 300 K after a 5 ns-long simulation using the ALIGN function implemented in Pymol^45^. We also performed the same analysis for the wild type enzyme (PDB code: 4WCT). The RMSD data are summarized in Table 5.

**Table 5:** Root Mean Square Deviation (RMSD) comparison for native enzyme, and mutants SS03 and SS17.

Mutant	RMSD [Å]
WT	0.99
SS03	0.93
SS17	1.35

The RMSD is calculated, for each enzyme between the crystallographic structure and end-point of 5 ns long MD simulation at 300 K. The RMSD calculation was done considering main chain atoms only.

Our results show that computational conformations are in very good agreement with the experimental structures. This demonstrates that our MD simulations are reliable and that the global conformations of the two mutants species are very similar to the native form of Amadoriase I.

Focusing on the region where the mutations were introduced, the SS03 mutant (S67C/P121C) is highly superimposable to the wild type enzyme (residues 62 to 72 and 116 to 126, RMSD = 0.842) while the SS17 variant (D295C/K303C) shows a structural rearrangement in the region from residue 290 to 308, with an RMSD of 1.657 Å (Fig. 8, Panel C and D).

Interestingly, while the introduction of the disulfide bond in the SS03 mutant does not cause any significant structural rearrangement in any portion of the enzyme, the formation of the disulfide bond in the SS17 mutant affects the conformation of one of the loops that define the boundaries of the tunnel leading to the catalytic pocket (residues 63 to 69) (Fig. 8, Panel B, orange circle). The RMSD between the SS17 mutant and the wild type enzyme for this short region is 2.745 Å. It is worth noting that despite this displacement, this portion of Amadoriase I is still structurally more stable than the corresponding loop in the crystal structure of Amadoriase II (PDB code: 3DJD), which could not be determined due to its high flexibility^9^.

## Discussion

Enhanced thermal stability is generally an essential requirement for those enzymes that are used as biocatalysts in industrial processes occurring at high temperatures. To this end, an enzyme can be modified by enhancing glycosylation^46,47^, or by introducing disulfide bridges^48^, salt-bridges^49^, or hydrogen bonds^50^. In particular, the introduction of disulfide bonds provide considerable stability to proteins by locking their fold in a well-defined local or global conformation^51,52^. Improved enzyme thermostability can usually be achieved either by structure-guided rational approaches^53^ or through a directed evolution campaign^54,55^. However, both these methods can be extremely time-consuming and highly labor-intensive. To the best of our knowledge, the only prior attempt to stabilize an Amadoriase enzyme has been carried out using a directed evolution approach that provided a *T*_50_ increase of ≈5 °C as a result of the combination of five point mutations^56^.

Here, with the goal of providing thermal stabilization to the Amadoriase I enzyme without incurring in an extensive experimental validation phase, we introduced a computational high-throughput screening method based on MD simulations, which is used to evaluate a library of mutations. Our library of potentially stabilizing disulfides was obtained with SSBOND code^39,40^. Based on the backbone and sidechain geometries of the WT, the software determined the residue pairs that, once mutated to cysteines, were likely to form disulfide bonds. This step led to the generation of 19 candidate enzymes, each with a different disulfide bond. We then simulated the WT and all 19 enzyme variants at three different temperatures (273, 310, and 340 K) and calculated the average RMSF at each simulated temperature. The slope (*λ*) of the linear interpolation of avg-RMSF as a function of the temperature was used as a proxy for enzyme stability. For most mutants *λ* was larger than that of the WT, suggesting that the introduction of those disulfide bonds would not be effective in reducing the thermal vibration that could eventually lead to local and then global unfolding. Conversely, four mutant models (SS03, SS07, SS11, and SS17) featured a reduced *λ*, indicating a possible stabilizing effect associated with the designed mutations.

The four mutants selected based on our computational filter were then expressed in *E.coli*, providing yields similar to the WT except for SS11, which appeared to be very poorly expressed and showed no binding to the FAD cofactor (Fig. 4), suggesting a crucial role for residues K233 and/or P404 in the folding of the enzyme.

After incubation with fructosyl-lysine at increasing temperatures, SS07 appears to be sligtly less active than the WT in the oxidized form, while showing stability levels comparable to those of the WT in the reduced form (Fig. 5, Panel C), suggesting a slightly detrimental effect associated with the introduction of the C106-C150 disulfide bond. Conversely, the SS03 mutant features a ~3 °C higher thermostability with respect to the WT (Fig. 5, Panel B). The SS17 variant, which shows an increase of ~8 °C in the *T*_50_ value and, contrary to all other tested mutants, a gradual decrease in residual activity at increasing temperatures, features detectable activity even after thermal treatment at 95 °C (Fig. 5, Panel D). To confirm the formation of the designed disulfide bond in the SS03 and in the SS17 mutants and to assess any possible change that such modifications may have caused to their global molecular architecture relative to the wild-type enzyme, we solved the crystal structures of these two thermostabilized species. Both structures were determined at high resolution (2.15 and 2.85 Å, respectively). Globally, both SS03 and SS17 maintain the same fold as the wild-type (RMSD 0.268 and 0.804 Å, respectively). However, while the SS03 mutant superimposes almost perfectly with the wild type in all the regions, displaying only a very minor twist in the loop hosting one of the mutated residues (S67C), SS17 shows a remarkable conformational change involving the region where the aminoacids that were mutated to cysteines in the SS03 mutant (S67 and P121) are found, the distance between these two aminoacids in the SS17 structure being 11.4 Å vs. 5.0 Å in the structure of the wild type enzyme. This might explain why, contrary to our expectations, the combination of the two stabilizing disulfide bonds (SS03 and SS17) in the SSDM species does not provide any further stabilization to the enzyme (Fig. 5, Panel E). However, this enzyme variant potentially carrying the double disulfide bonds appears to perform similarly to the SS03 variant, possibly suggesting that, in the course of the SSDM folding process, long range effects result in significantly pronounced deviations in the final enzyme conformation, thus likely allowing the formation of the C67-C121 disulfide bond while preventing the C295-C303 to form. Unfortunately, we could not obtain the structure of the SSDM species due to poor quality crystals. However, although we cannot be sure whether both disulfide bonds did indeed form in our double mutant variant, a feature that, as speculated, appears unlikely based on the crystal structure of the two single mutants that we have determined, our enzymatic data do rule out any possible cooperative effect associated with the simultaneous presence of these four specific cysteines in the sequence.

It should be noted that the proposed computational screening method produced two false positives: the SS11 variant does not fold under standard expression conditions, while SS07 shows a decreased enzymatic activity relative to the wild type form of the enzyme. In the case of SS11, we hypothesize that the mutations are in a critical position for the correct folding of the enzyme due to their proximity to the cofactor. Hence, they may affect the extremely conserved fold around the cofactor region. However, improper folding cannot be predicted with MD, and this is certainly a general limitation of any computational screening method. The SS07 variant, albeit correctly folded and active, shows reduced activity towards its natural substrate. In this case, the disulfide bond is located close to the entry tunnel and produces a narrowing effect that possibly hampers accessibility to the active site of the enzyme. In general, it is not surprising that our screening analysis produces a certain number of false positives. However, we believe that our computational screening method, especially in the initial phases of an enzyme design campaign, still provides a fast and efficient way to identify candidate active mutants while limiting the experimental burden and costs.

## Conclusions

Using a rational *in silico* computational design and screening method, we have produced two forms of the deglycating enzyme Amadoriase I featuring a significantly higher thermostability compared to the WT enzyme. In particular, the SS17 variant represents a possible starting point for the development of improved biosensors to be used for the detection of diabetes, for the design of therapeutic tools aimed at the prevention or the reduction of protein glycation in biological tissues, and as a potential tool for the reduction of acrylamide formation in food processing. The proposed computational screening method appears to be a promising tool to expedite the design of thermostable enzymes. Compared to directed evolution, the method used herein allows for a significant reduction in cost and time for the experimental production and characterization of candidate thermostable enzyme variants. Future work will be aimed at testing this methodology on other type of mutations beyond disulfide bonds, and extending the validation of our screening strategy to other enzymes.

Concerning glycated protein detection, stabilized enzymes may improve the typical shelf-life of the biosensors, of which the enzymes are usually the most sensitive component.

The application of Amadoriases in the food industry as a tool for acrylamide reduction requires that the enzyme be compatible with the high temperatures that are commonly associated with food treatments. High temperatures are known to facilitate the formation of Amadori products, which in some cases are undesired as they change the organoleptic as well as the nutritional profile of foods, often resulting in potentially harmful byproducts (as asparagine glycation, which turns into acrylamide). For example, roasting requires temperatures from 204 °C and above, frying in the range of 177–191 °C, while baking occurs at oven temperatures around 250 °C. On the other hand, there are some other thermal treatments, such as those related to milk conservation that take place at lower temperatures. For example, high-temperature short-time (HTST) milk pasteurization is done at 72 °C for 15 seconds, while milk ultra-heat treatment (UHT) is done at 140 °C (284 °F) for four seconds. Also, other treatments related to milk conservation occur within a temperature range from 63 °C for 30 minutes (the typical FDA-approved home pasteurization treatment) to 71.7–72 °C for 15 seconds (UK Dairy Products Hygiene Regulations 1995). For these applications, the increase in T_50_ shown by our mutants may well represent a step forward in the direction of obtaining a stable and useful enzyme for glycosylation prevention.

Finally, the use of Amadoriases to prevent protein glycation *in vivo* will require extensive mutagenesis and reduction of secondary structure elements to provide larger access to the catalytic site. Owing to the likely destabilization effect that is expected to be associated with such extensive structural modifications^57^, Amadoriases with enhanced stability will more likely represent more suitable candidates for the introduction of large yet sustainable structural changes in the enzyme.

## Material and Methods

### Computational design of disulfide bonds

To identify suitable positions for the introduction of disulfide bonds, we used the disulfide bond prediction program SSBOND^39^. The 1.67 Å crystal structure of the wildtype (WT) enzyme Amadoriase I^41^ (PDB code: 4XWZ) was used as a template. To avoid mutations that may affect enzymatic activity, we allowed only the substitution of those residues that are located farther than 10 Å from the FAD cofactor or from the ligand position. SSBOND allows the identification of potential disulfide bond sites based on conformational and energy constraints determined from disulfide bond-containing protein structures that are deposited in the PDB. Using an energy cut-off of 10 kcal/mol, the program generated a putative list of 19 pairs of residues that, if mutated to cysteines, would allow the introduction of stabilizing disulfide bonds in the structure of the enzyme. The output of the program includes the dihedral angles that describe the disulfide bond conformation and an energy rank that estimates the conformational strain thus introduced.

### Molecular Dynamics simulations

We tested the 19 Amadoriase I variants (named SS01 to SS19), each with a different disulfide bond, using molecular dynamics (MD) simulations and we derived those putative sites where the introduction of an SS bond may enhance protein stability. The 19 molecular models were minimized and equilibrated following protocols used in previous studies^44,58–60^. Specifically, each variant was modelled using the AMBER99SBildn force field^61^ for protein, water (TIP3P) and ions, while the FAD cofactor was modelled using the general amber force field (GAFF)^62^. The enzyme models were then solvated with ≈14,992 TIP3P water molecules. Since the enzyme carries a net charge (−10 for the WT, while a slightly different one for each variant depending on the mutation), ions (specifically, Na^+^ and Cl^−^) were added to neutralize the system and provide a physiological ionic strength of 0.15 mol/L. The setup resulted in systems consisting of ≈50.000 atoms in a simulation box with initial dimensions of approximately 70 × 70 × 85 Å^3^.

Using NAMD code^63^, the systems were first minimized for 2,000 steps using the conjugate gradient algorithm. Then, they were equilibrated in NPT ensemble at 1 atm and at three different temperatures for each variant (273, 300, and 340 K, respectively). Langevin dynamics was used to maintain constant temperature with a coupling coefficient of 1 ps^−1^, and pressure was maintained using the Nosé-Hoover method. In the NPT simulations, we used a time step of 2 fs, a non-bonded cut-off of 9 Å, rigid bonds and particle-mesh Ewald long-range electrostatics. During minimization and NPT equilibration, the C_α_ atoms of the protein were restrained by a 10 kcal mol^−1^ Å^−2^ spring constant to prevent protein conformation to be disrupted in the initial phase of system equilibration. Finally, the production runs were done using ACEMD^64^ and a time step of 4 fs, while all the other parameters (non-bonded cut-off, and PME) were maintained as in the equilibration phase. We simulated each variant for 5 ns at the three different temperatures (273, 300, and 340 K) in explicit solvent. The Root Mean Square Displacement (RMSD) was monitored to confirm the stability of the simulations (see Fig. S4 in Supplementary Information).

### Computational screening of mutants

**Figure 9:**
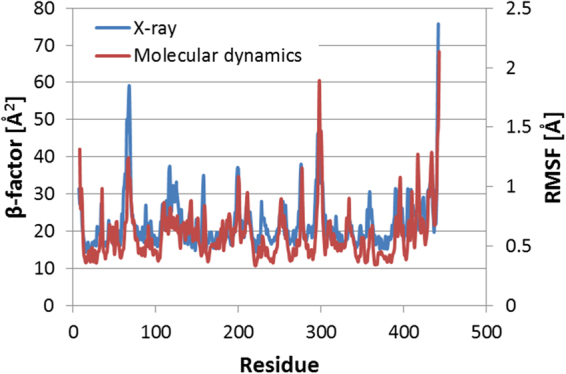
Superposition of the RMSF of WT-Amadoriase I and experimental β-factor, showing how the RMSF from MD simulations reproduces the same trend of the experimental β-factors and it allows to pinpoint the same peaks, which represent highly flexible protein regions.

In order to define a quantitative mutation screening criteria, we used the Root Mean Square Fluctuation (RMSF), which correlates with the β-factor from the X-ray crystal structures^65^ (Fig. 9). The RMSF was calculated over the MD trajectory (excluding the first ns) for the C_α_ of the protein from from residue 10 to 437 (we excluded the N- and the C-terminus of the protein due to their intrinsic high mobility).

The RMSF was then calculated at increasing temperatures (273, 300, and 340 K). As shown in Fig. 2, Panel A, the RMSF baseline increases with the temperature due to higher kinetic energy. However, the RMSF increases significantly more in specific regions of the protein, which may be assumed to represent the origin of local protein unfolding. For each temperature, we calculated the average RMSF (avg-RMSF) and we calculated the slope (*λ*) of avg-RMSF vs. temperature (Fig. 2, Panel B and Fig. S3 of Supplementary Information). The value of *λ* represents an estimate of the increase of enzyme flexibility with the temperature. If an SS-variant showed a *λ* higher than the one calculated for the WT, the proposed SS-mutant was rejected. Otherwise, if *λ* was lower than that of WT, the enzyme variant was regarded as more thermostable than the WT and hence selected for experimental validation.

In order to test the effect of the simulation length, we performed 50 ns long MD simulations (at temperature of 273, 300 and 340 K) for the wild type enzyme and for the variants SS03, SS07, SS11, and SS17. We observed that all the structures reached stability within the first ns (see Fig. S5 in Supplementary Information), also at the highest simulated temperature. Furthermore, we compared the conformation at 300 K for the 5 ns MD simulations with the conformation obtained with 50 ns MD simulations, showing negligible differences (see Fig. S6 in Supplementary Information). The RMSD difference between the conformations after 5 ns and after 50 ns is approximately 1 Å in all cases (see Table S1 of SI).

In addition, increasing the simulation length from 5 ns (Fig. S7A) to 50 ns (Fig. S7B) did not produce significant changes in the results in terms of λ parameter (see also Table S2). Moreover, while introducing extra computational costs, the inclusion of simulations at higher temperatures (e.g., 380 K) did not alter the results (see Fig. S7C).

### Materials

DNA primers were from Metabion International AG. The gene sequence coding for Amadoriase I was cloned in a pET-3a vector (pET3a-AmadI) in our laboratory, and the cloning procedure has been described elsewhere^41^. Fructosyl-lysine synthesis and characterization is described in the Supplementary Information. All the chemicals were from Sigma-Aldrich, unless otherwise indicated, and were of the highest commercial purity available (>95%).

### Protein expression and purification

The point mutations required for the selected double-cysteine mutants were introduced by PCR in the pET3a-Amad I using the QuikChange II site-directed mutagenesis kit (Agilent) according to the manufacturer’s protocol. The generated mutants and the used primers are shown in Table S3 of Supplementary Information. The resulting mutant plasmids were analyzed and validated by DNA sequencing. All the proteins used in the present study were expressed in *E. coli* BL21(DE3)pLysS cells (Invitrogen) and purified following the same protocol. Cells were grown at 37 °C in 1 liter of Luria-Bertani (LB) broth, supplemented with 50 mg/liter ampicillin until they reached an OD_600_ = 0.6. After induction with isopropyl 1-thio-β-D-galactopyranoside (IPTG) to a final concentration of 0.5 mM, the bacteria were cultured overnight at 25 °C. Cells were then harvested by centrifugation and resuspended in Buffer A (50 mM Tris-HCl pH 7.4, 150 mM NaCl) supplemented with a protease inhibitor cocktail and DNAse and then lysed by sonication on ice. The lysate was clarified by centrifugation and the soluble fraction was loaded onto a Ni-NTA (Qiagen) column equilibrated with Buffer A. The Ni-NTA beads were first washed using 5 column volumes of Buffer A supplemented with 40 mM imidazole, then the bound N-terminal His-tagged enzyme was eluted with same buffer supplemented with 400 mM imidazole. The positive fractions were pooled, concentrated using an Amicon 20 centrifugal filter with a molecular weight cut-off of 10 KDa and loaded onto a Superdex 200 Increase 10/300GL size exclusion column (GE Healthcare) pre-equilibrated with Buffer B (10 mM Tris buffer pH 8.0). The resulting protein was collected, concentrated to ≈10 mg/mL and stored at −80 °C. Protein concentration was assessed using a Bradford assay^66^ kit (Bio-Rad) and bovine serum albumin (Sigma) as the standard. Sample purity was assessed by 10% SDS-PAGE.

### Absorption spectra

All spectra were recorded at 25 °C in a solution of Buffer B, containing protein concentrations ranging between 1 and 10 μM, with a Tecan Spark10 M and normalized to the absorbance at 280 nm.

### Enzyme activity assay

Enzymatic activity was followed by a continuous assay that detects glucosone formation over time from fructosyl-lysine at 322 nm, as previously described^41^. The assay was adapted to support high throughput format in a 96 transparent polystyrene plates from Grainer Bio. The 200 μL reaction mixture contained 10 mM Tris HCl pH 7.4, 20 mM o-phenylenediamine, 2 mM fructosyl-lysine. After 1 minute of pre-incubation, the reaction was started by adding the enzyme at a final concentration of 0.44 µM, and the increase in absorbance at 322 nm (glucosone ε_322_ = 149.25 M^−1^ cm^−1^) was monitored in a Spark10 M (Tecan). Unless otherwise indicated, enzymatic activity was assayed at 25 °C. One unit (U) is defined as the amount of enzyme required to produce 1 µmol of glucosone per minute, and specific activity was expressed as U mg^−1^ of enzyme.

### Steady-State kinetics

Apparent steady-state kinetics measurement for all the enzymes over its natural substrate were determined by means of the assay described above, with fructosyl-lysine concentrations varying from 0.05 to 2 mM. Data points were obtained from three independent experiments. Data were fitted by non-linear least-square fit of the data, with Eq. 1 (the Michaelis-Menten equation for hyperbolic substrate kinetics) using Hyperbola fit function of GraphPad Prism version 5.00 for Windows, GraphPad Software, La Jolla California USA.

1 $${v}=\frac{{{V}}_{{\max }}\ast {S}}{({{K}}_{{m}}+{S})}$$

in which *v*, *V*_*max*_, *S*, and *K*_*m*_ represent the steady state reaction rate, maximum reaction rate, substrate concentration, and Michaelis-Menten constant for the substrate, respectively.

### Characterization of the thermoresistant mutants

Thermal stability tests were performed using the assay described above after heat treatment, by incubating for 10 minutes the enzyme to the target temperature ranging from 25 to 100 °C (with 5 °C steps) in Buffer B, in the absence of ligands, and then cooling it down at 4 °C until test. The reduced forms of the enzymes were obtained by supplementing the buffer with 100 mM 1,4-dithiothreitol (DTT). After 1 h of incubation, the heat treatment and enzymatic assay were performed as for the oxidized forms. The *T*_50_ values were obtained by fitting the data with the Boltzmann equation (Eq. 2) using the Boltzmann sigmoidal fit function implemented in GraphPad Prism version 5.00 for Windows, GraphPad Software, La Jolla California USA.

2 $$A={A}_{bottom}+\frac{({A}_{top}-{A}_{bottom})}{1+{e}^{(\frac{T-{T}_{50}}{s})}}$$

where *A* represents the residual activity, *A*_*bottom*_ the lower asymptote of residual activity, *A*_*top*_ the higher asymptote of residual activity, *T* the temperature, *T*_*50*_ the temperature at which residual activity is halfway between *A*_*top*_ and *A*_*bottom*_, and *s* the steepness of the curve.

The optimal pH for the WT and SS-mutants was determined by measuring the enzyme activity using the same assay from pH 6.0 to pH 11.0 with the following buffers (10 mM): Sodium Citrate (pH 6.0); Tris-HCl (pH 7.0, 8.0 and 9.0); CAPS-NaOH (pH 10.0, 11.0).

For all the listed experiments, data points were obtained from three independent experiments.

### Protein crystallization and Structure determination

Crystals of both the SS03 and the SS17 mutant were obtained using the vapor diffusion method at room temperature by mixing a 1 μL drop of ~15 mg/mL protein sample with an equal volume of a 0.1 M sodium citrate pH 5.6, 14% PEG4K, 15% isopropanol and 0.1 M sodium citrate pH 5.6, 14% Peg4K, 5% dimethyl sulfoxide solution respectively. Medium-size (150 × 100 × 50 µm) rod-like crystals appeared within a few days. Prior to X-ray data collection, crystals were frozen in a chemically identical solution supplemented with 25% (v/v) glycerol for cryo-protection. A 2.19 Å resolution data set and a 2.85 Å resolution data set were collected from a crystal of SS03 and a crystal of SS17, respectively, in both cases using λ = 1.000 Å in the X06DA-PXIII beamline at the Swiss Light Source (Paul Scherrer Institute, Villigen, Switzerland). Diffraction images were processed and scaled using XDS^67^. The structures were determined by molecular replacement using MOLREP^68^ from the CCP4 package^69^ and the free Amadoriase I structure (PDB code: 4WCT) as the search probe. Model building and refinement were carried out using REFMAC5^70^ and PHENIX^71^. Water molecules were added both automatically using the phenix_refine tool from the PHENIX package and manually from visual inspection of the electron density map. All the figures in the paper were generated using PyMOL^45^ or VMD^72^

**Table 6:** Data collection and refinement statistics of the SS03 and the SS17 crystal structures.

Crystal	SS03 (PBD id: 5OC3)	SS17 (PBD id: 5OC2)
**Data collection** Space group Cell dimensions *a (Å)* *b (Å)* *c (Å)* β (°) Wavelength (Å) Resolution (Å) *R*_*sym*_ *or R*_*merge*_ *(%)* *I/σI* Completeness (%) Multiplicity	P2_1_2_1_2_1_ 70.157 83.081 175.795 1 47.87–2.15 9.4 (31.1) 15.95 (6.3) 99.9 (99.7) 6.8	P2_1_ 68.853 90.462 81.307 102.89 1 48.82–2.85 16.5 (49.5) 10.6 (4.1) 100.0(100.0) 6.9
**Refinement** Resolution (Å) No. of reflections *R*_*work*_/*R*_*free*_ *(%)* No. of atoms Protein Ligand: FAD-FLY Water Average B-factors (Å^2^) Protein (chain A – chain B) Ligand Water r-m-s-d Bond lengths (Å) Bond angles (°) Ramachandran Most favoured (%) Additional allowed (%) Disallowed (%)	47.87–2.15 56474 13.92/18.07 6935 106 923 10.66–12.26 5.17 23.24 0.009 1.173 97.96 2.04 0.0	46.37–2.85 22838 17.18/24.40 6900 106 282 11.83–13.15 7.31 8.89 0.008 1.23 95.79 3.87 0.34

. The refinement of the SS03 and the SS17 structures converged to a final R/R_free_ = 13.92/18.07% and 17.18/24.40%, respectively. Data collection and final refinement statistics are shown in Table 6.

### Accession Numbers

The final crystallographic coordinates of the crystal structures shown here are available in the RCSB PDB (accession code: 5OC2 and 5OC3).

## Electronic supplementary material

Supplementary information
