# Supplementary material for: Thermal stabilization of the deglycating enzyme Amadoriase I by rational design
Source: Sci Rep. 2018 Feb 14;8:3042. doi: 10.1038/s41598-018-19991-x (PMC5813194; doi:10.1038/s41598-018-19991-x)
Supplement: Supplementary file 1 — Supplementary information [file 41598_2018_19991_MOESM1_ESM.pdf]

Supplementary information

Thermal stabilization of the deglycating enzyme Amadoriase I by rational design.

Federica Rigoldi[1,ǂ], Stefano Donini[2,ǂ], Francesca Giacomina[3], Federico Sorana[3], Alberto Redaelli[1], Tiziano Bandiera[3], Emilio Parisini[2,*] , Alfonso Gautieri[1,*]

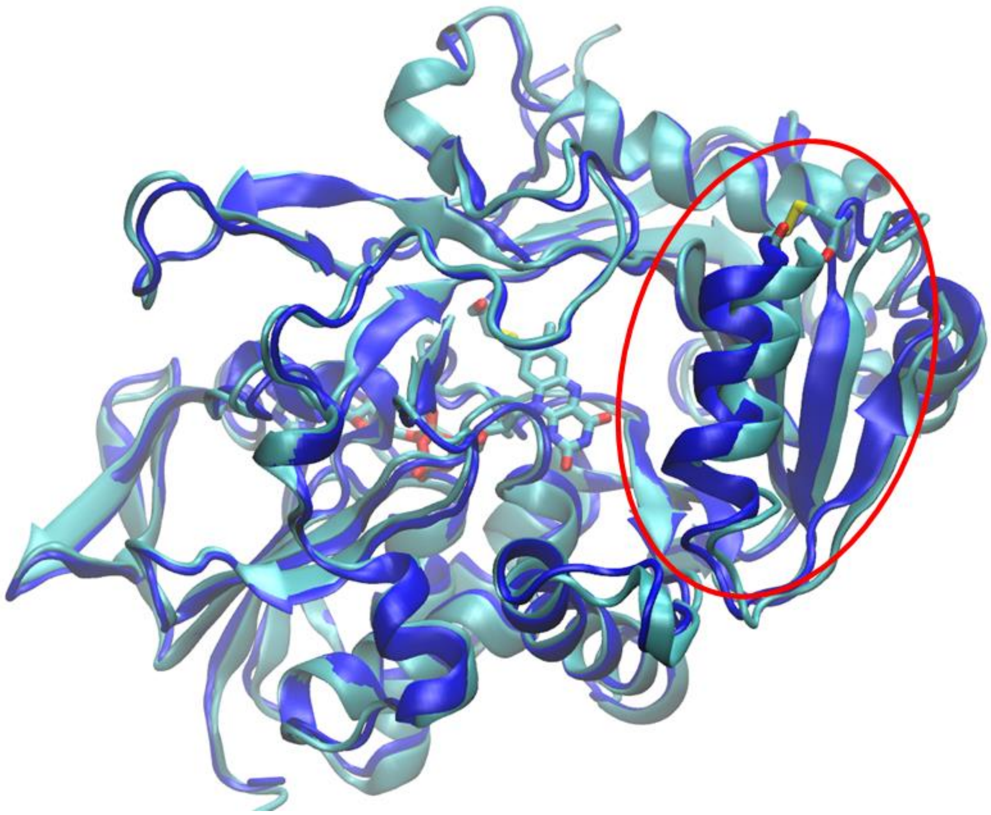

Figure S1. Superposition of WT (light blue) and SS07 (blue) at the end of MD simulation at 300K. *The figure shows that there is non-negligible structural difference between WT and SS07 in the region close to the introduced disulphide. Since this region is also defining the entrance to the tunnel leading to the catalytic site, it is possible that the introduced mutations are detrimental for the enzymatic activity.*

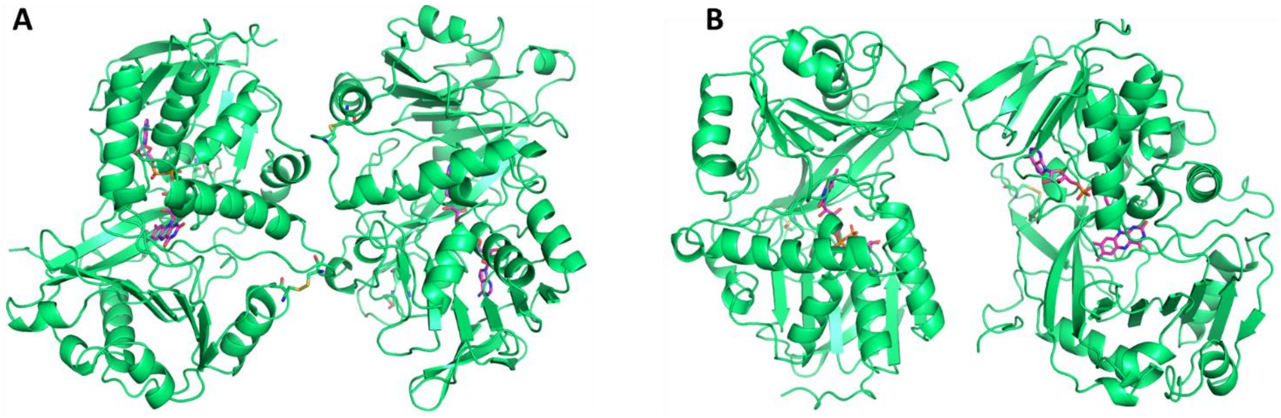

Figure S2. *Different packing arrangements of the two independent molecules in the SS03 mutant (Panel A) and in the SS17 mutant (Panel B).*

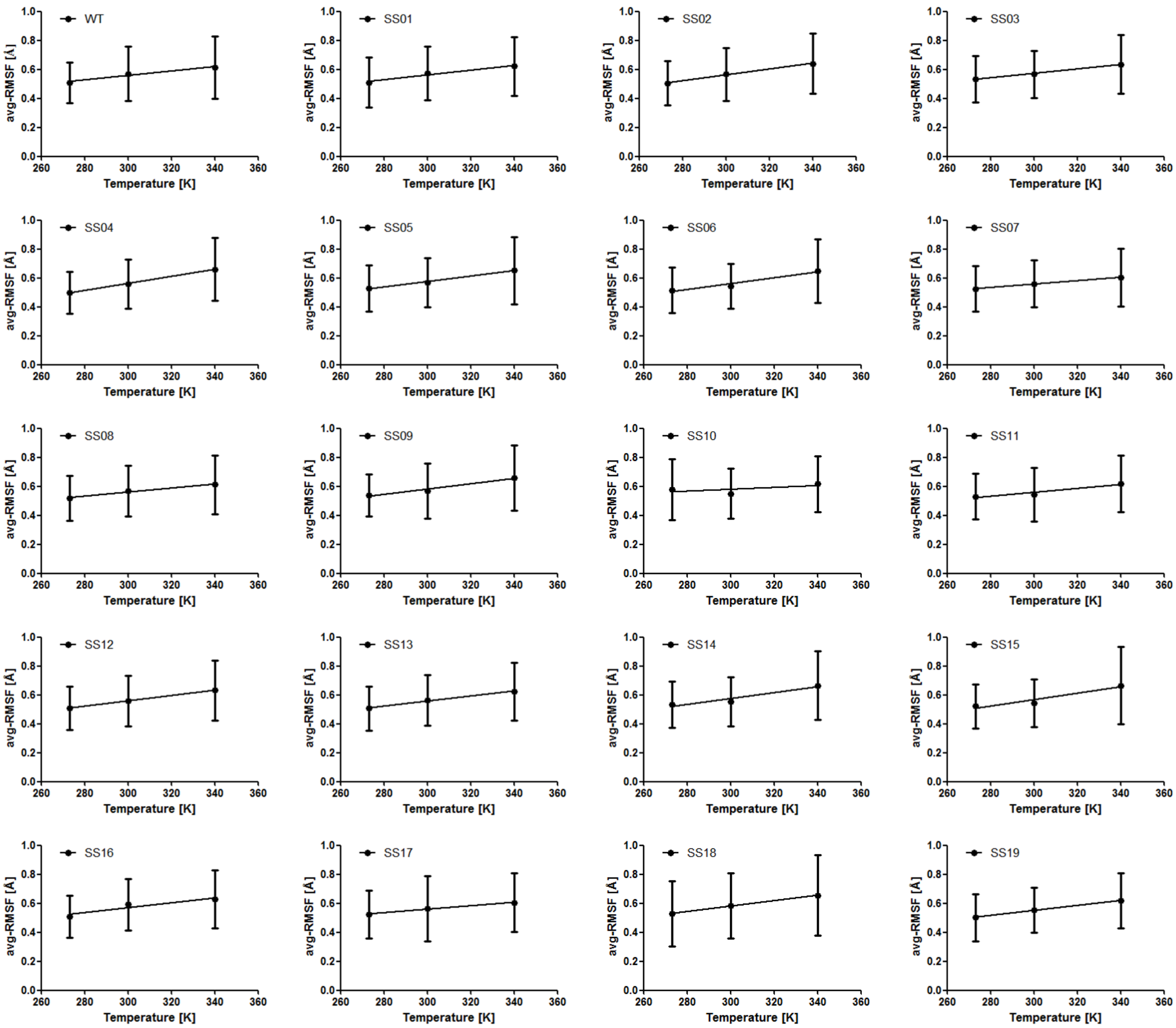

Figure S3. *Plot of avg-RMSF vs. Temperature for all the screened Amadoriase variants.*

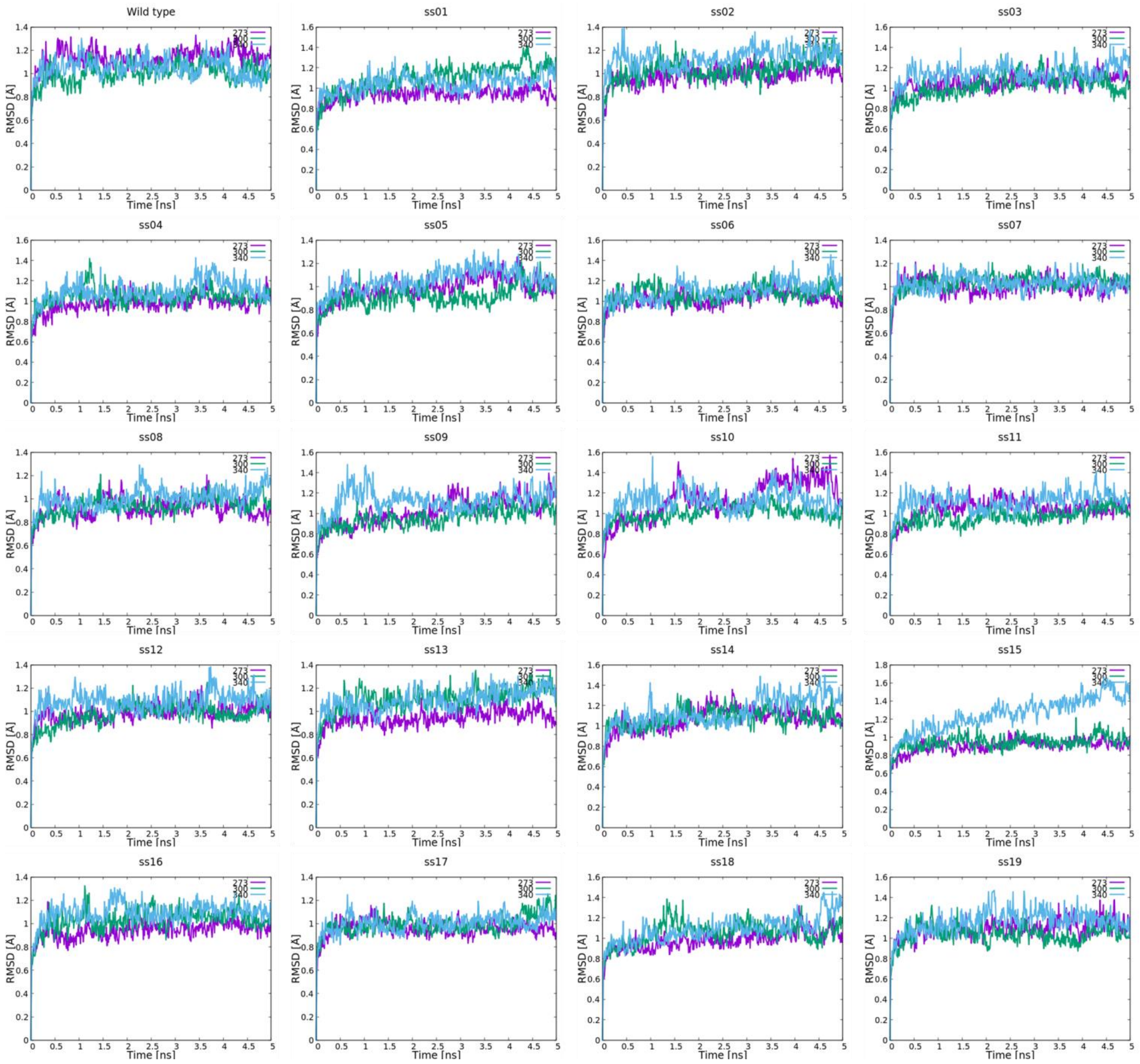

Figure S4. RMSD vs. simulation time. *Each panel shows the RMSD of the enzyme variants as a function of the simulation time. Simulations at T=273K are represented by purple lines, simulations at T=300K are represented by green lines and simulations at T=340K are represented by blue lines.*

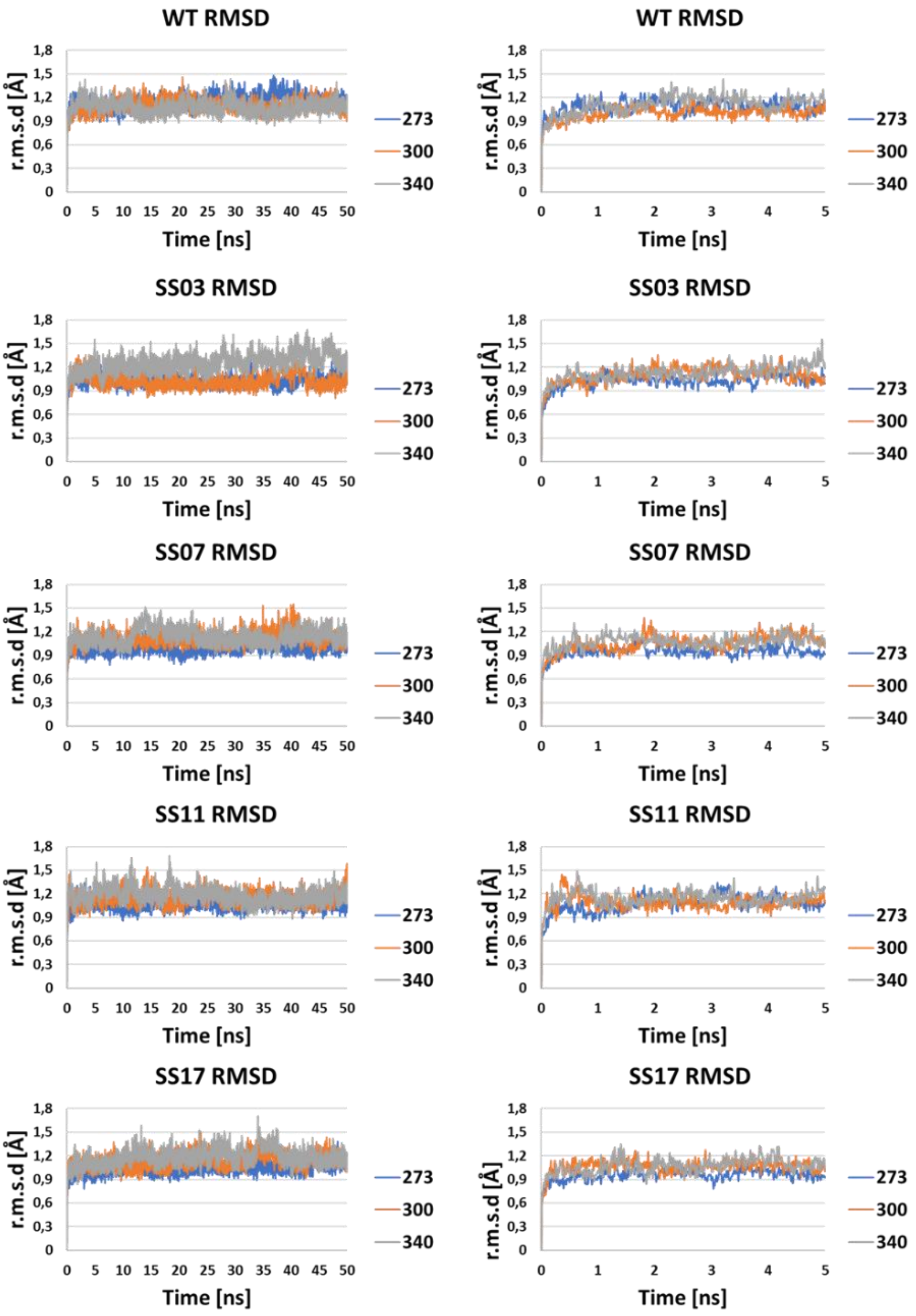

Figure S5. *RMSD trend of wild-type enzyme and four relevant mutants, at the three simulated temperatures (273, 300, 340 K). Results for the 50 ns MD simulations are shown in the left panels, while results for the 5 ns MD simulations are shown in the left panels. For all tested mutants, the RMSD reach stability within the first nanosecond of MD simulations.*

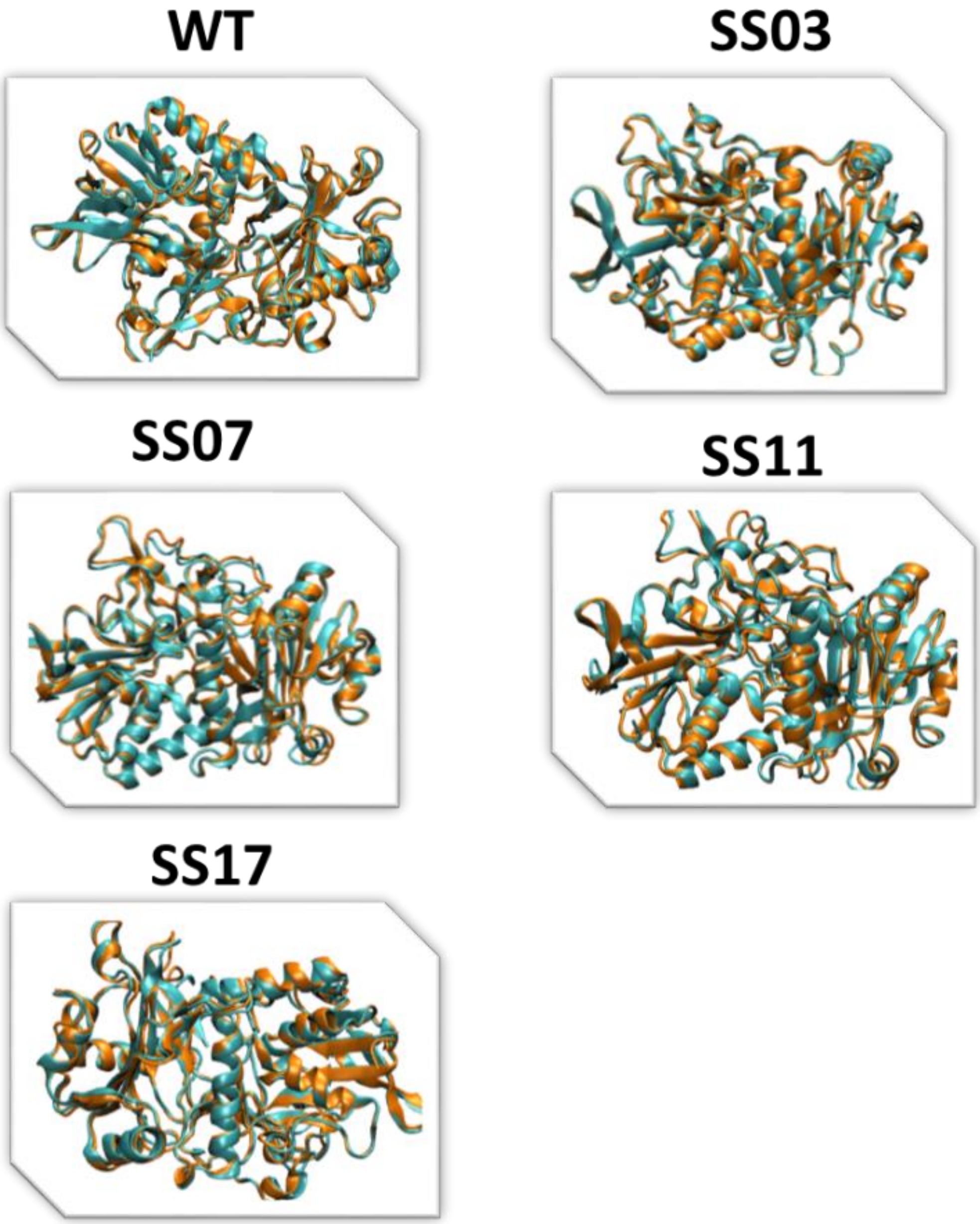

Figure S6. *Superposition of final structure of the 5 enzymes variants after the simulation at 300K for 5 ns (cyan) and after 50 ns simulation (orange).*

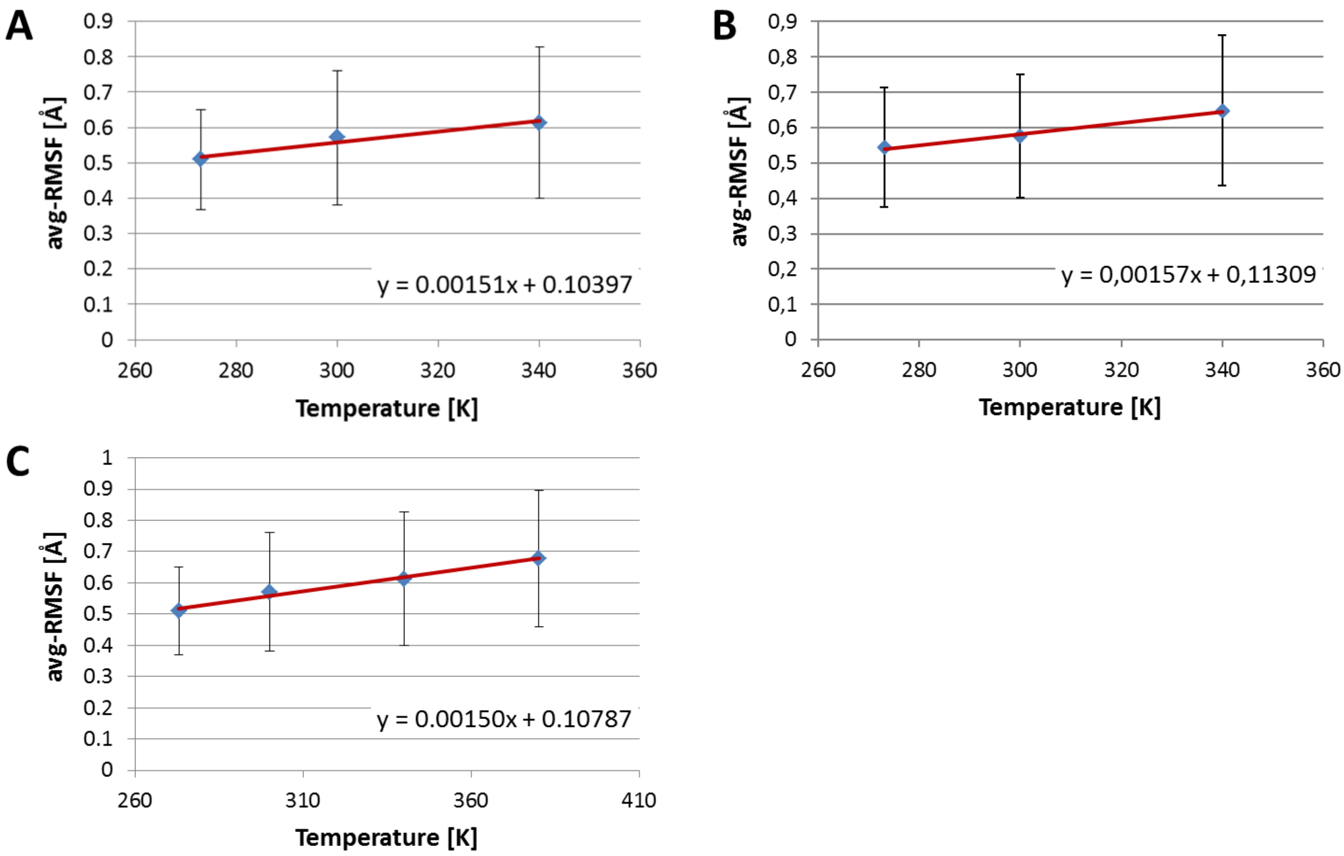

Figure S7. In silico screening tests. *Results are shown for the WT, the disulphide variants present homologous trends. Panel A shows the average RMSF of the WT enzyme calculated for 3 different temperatures over 5 ns simulations. We also tested whether longer simulations (Panel B, simulations of 50 ns) or more data points would be required (Panel C, extra data at 380K). The results show that extending simulation time or including simulations at 380 K does not alter the results while increasing computational costs significantly.*

Enzyme	RMSD[Å]<br>Backbone only<br>5ns-50ns	RMSD [Å]<br>Incl. side chains<br>5ns-50ns
**WT**	0.957	1.415
**SS03**	0.967	1.370
**SS07**	0.977	1.472
**SS11**	1.124	1.547
**SS17**	0.917	1.400

Table S1. *RMSD comparison for resulting structures at the end of 5ns and at the end of 50ns MD simulations.*

Enzyme	Mutated residues	5 ns MD		50 ns MD	
		**λ**	R^2	**λ**	R^2
WT	-	*0.00151*	*0.953*	*0.00157*	*0.989*
SS03	S67, P121	0.00151	0.995	*0.00158*	*0.932*
SS07	H106, G150	0.00115	0.997	*0.00112*	*0.975*
SS11	K233, P404	0.00134	0.924	*0.00140*	*0.977*
SS17	D295, K303	0.00118	0.993	*0.00123*	*0.963*

Table S2. *Comparison of results obtained from 5 ns MD simulations and results obtained with 50 ns MD simulations.*

Mutant	Mutation	Primers
**SS03**	S67C	Forward GAACTGAAAGATGGCTGTAGTGATCCACGCTCT<br>Reverse AGAGCGTGGATCACTACAGCCATCTTTCAGTTC
	P121C	Forward GATGAAGTTGAATGTTCTGAAACCAACTTCGTGAAACTCGAG<br>Reverse CTCGAGTTTCACGAAGTTGGTTTCAGAACATTCAACTTCATC
**SS07**	H106C	Forward TTCATCATCAGCGGTTGTACACCGGCTCTGATT<br>Reverse AATCAGAGCCGGTGTACAACCGCTGATGATGAA
	G150C	Forward ACAGGGGACTTTCCGTGTTGGAAAGGCTGGTTG<br>Reverse CAACCAGCCTTTCCAACACGGAAAGTCCCCTGT
**SS11**	K233C	Forward GATTCCCTGTTAGACTTTTGTAAGCAGTTACGTCCCACC<br>Reverse GGTGGGACGTAACTGCTTACAAAAGTCTAACAGGGAATC
	P404C	Forward ATTGTCCGTTGGCGTTGTGAAACCGCGGTTGAT<br>Reverse ATCAACCGCGGTTTCACAACGCCAACGGACAAT
**SS17**	D295C	Forward GGGTATTGCAACTTTCTCCCATGTCCGAATCGTCCCGG<br>Reverse CCGGGACGATTCGGACATGGGAGAAAGTTGCAATACCC
	K303C	Forward CGAATCGTCCCGGTCAAGAGTGTAGCGTCCCGTTCGC<br>Reverse GCGAACGGGACGCTACACTCTTGACCGGGACGATTCG

Table S3. *Forward and reverse primers used for double point mutations. Underlined codons correspond to the mutated residues*

Fructosyl lysine (FruK) synthesis.

Solvents and reagents were obtained from commercial suppliers and were used without further purification. The synthesis followed the procedure summarized in figure S3.

List of abbreviations: acetonitrile (MeCN), chloroform-*d* ($CDCl_3$), copper chloride (CuCl), deuterium oxide (D_2O), dichloromethane (DCM), diethyl ether (Et_2O), diisopropyl azodicarboxylate (DEAD), dithiotreitol (DTT), ethanol (EtOH), ethyl acetate (EtOAc), methanol (MeOH), *N,N*-dimethylformamide (DMF), palladium on charcoal (Pd/C), pyridine (py), sodium hydroxide (NaOH), sodium metoxide (NaOMe), sodium sulphate (Na_2SO_4), *tert*-butanol (*t*-BuOH), tetrahydrofuran (THF), thioacetic acid (AcSH), total ion current (TIC), triethylamine (Et_3N), triflic anhydride (Tf_2O), trifluoroacetic acid (TFA), triphenylphosphine (PPh_3).

Automated column chromatography purifications were performed on Teledyne ISCO apparatus (CombiFlash® Rf) with pre-packed silica gel columns of different sizes (Redisep). NMR experiments were run on a Bruker Avance III 400 system (400.13 MHz for ^{1}H and 100.62 MHz for ^{13}C), equipped with a BBI probe and Z-gradients, and on a Bruker FT NMR Avance III 600 MHz spectrometer equipped with a 5 mm CryoProbeTM QCI $^{1}H/^{19}F–^{13}C/^{15}N–D$ quadruple resonance, a shielded z-gradient coil and the automatic sample changer SampleJet™ NMR system (600 MHz for ^{1}H, 151 MHz for ^{13}C, and 565 MHz for ^{19}F). Chemical shifts for ^{1}H and ^{13}C spectra were recorded in parts per million using the residual non-deuterated solvent as the internal standard [for $CDCl_3$: 7.26 ppm, ^{1}H, and 77.16 ppm, ^{13}C; for DMSO-d_6: 2.50 ppm, ^{1}H, 39.52 ppm, ^{13}C; for D_2O: 3-(trimethylsilyl)-2,2,3,3-tetradeuteropropionic acid (TSP-d_4) as internal standard, 0.00 ppm]. UPLC-MS analyses were run on a Waters ACQUITY UPLC-MS system consisting of a SQD (Single Quadrupole Detector) Mass Spectrometer equipped with an Electrospray Ionization interface and a Photodiode Array Detector (PDA). PDA range was 210-400 nm. Electrospray ionization in positive and negative mode was applied. Mobile phases (A) 10 mM NH_4OAc in H_2O, pH 5; (B) 10 mM NH_4OAc in $MeCN/H_2O$ (95:5) pH 5. Analyses were performed with method A.
Method A
Gradient: 5 to 95% B over 3 min. Flow rate 0.5 mL/min. T 40 °C
Pre column: Vanguard BEH C_{18} (1.7 µm 2.1 x 5 mm). Column: BEH C18 (1.7 µm 2.1 x 50 mm)

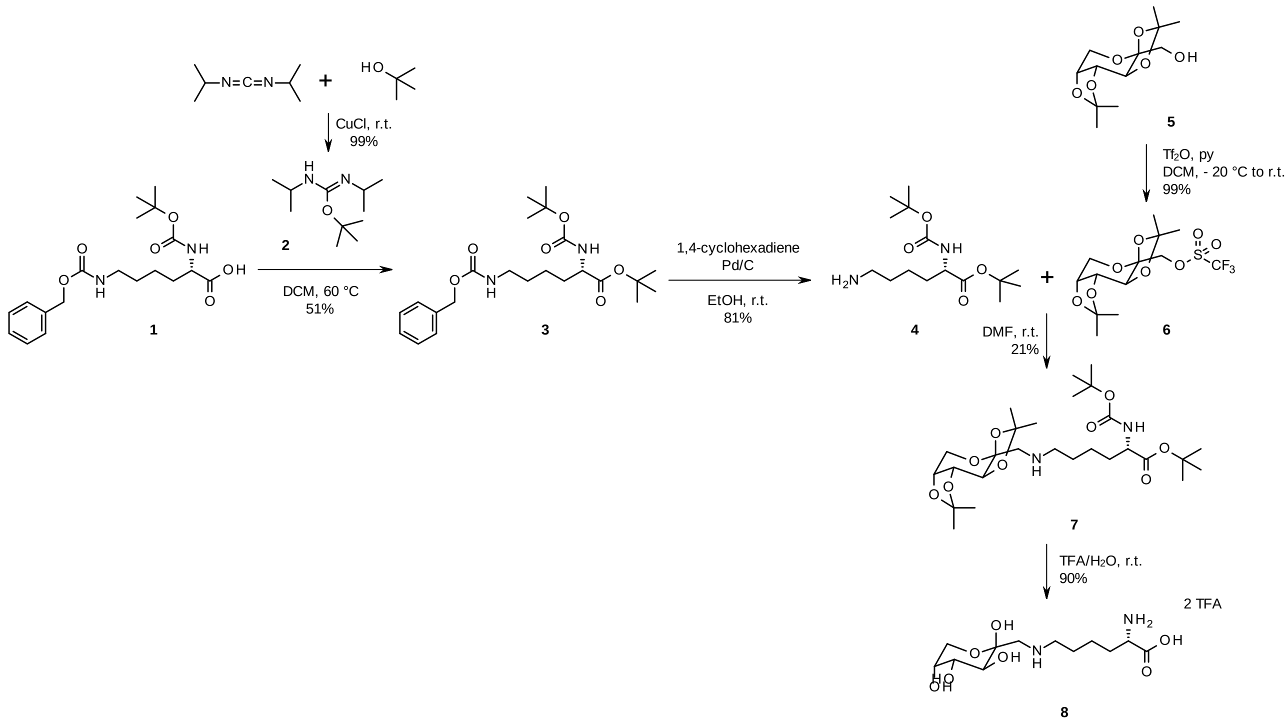

Figure S3. *Synthetic route to Fructosyl lysine (FruK)*

2-*tert*-Butyl-1,3-diisopropyl-isourea (2)

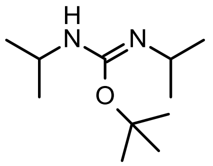

Copper (I) chloride (0.18 g, 0.01 eq., 1.81 mmol) was added to a solution of N,N'-diisopropylmethanediimine (28 mL, 1.0 eq., 181 mmol) in dry *t*-BuOH (19.9 mL, 1.15 eq., 208 mmol). The reaction mixture was stirred at room temperature for 14 h, then filtered and concentrated under reduced pressure. The residue was used in the next step without further purification.

^{1}H NMR (400 MHz, $CDCl_3$-*d*): δ 3.80–3.64 (m, 1H), 3.32–3.06 (m, 1H), 1.64–1.42 (m, 9H), 1.21–1.04 (m, 12H).

***tert*-Butyl (2*S*)-6-(benzyloxycarbonylamino)-2-(tert-butoxycarbonylamino)hexanoate (3)**

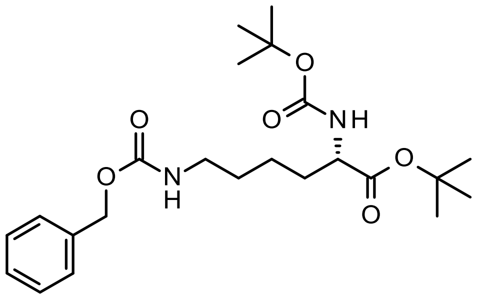

To a solution of (2*S*)-6-(benzyloxycarbonylamino)-2-(tert-butoxycarbonylamino)hexanoic acid (**1**) (5.0 g, 1.0 eq., 13.14 mmol) in DCM (50 mL), **2** (5.26 g, 2.0 eq., 26.28 mmol) was added. The reaction mixture was heated at reflux for 3 h, then cooled to room temperature and concentrated *in vacuo*. The residue was filtered through a short pad of silica eluting with EtOAc, and further purified by flash chromatography eluting with cyclohexane/EtOAc (0 to 100%) to give the pure title compound as colourless oil (2.923 g, 51%). UPLC-MS (method A): Rt. 2.67 min; ionization ES^+ 437 $[M+H]^+$.

^1H NMR (400 MHz, $CDCl_3$-*d*): δ 7.41–7.31 (m, 5H), 5.12 (s, 2H), 5.09–5.04 (m, 1H), 4.91–4.75 (m, 1H), 4.25–4.11 (m, 1H), 3.22 (q, *J* = 6.6 Hz, 2H), 1.85–1.74 (m, 1H), 1.59 (s, 12H), 1.50–1.42 (m, 11H).

tert-Butyl (2*S*)-6-amino-2-(tert-butoxycarbonylamino)hexanoate (4)

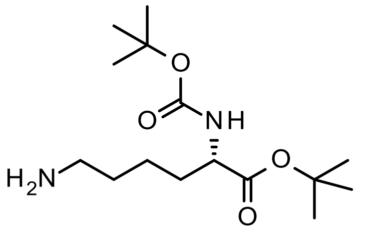

Under nitrogen atmosphere, a solution of **3** (2.92 g, 1.0 eq., 6.7 mmol) in EtOH (200 mL) was initially stirred, followed by subsequent addition of 10% Pd/C (3.0 g) and 1,4-cyclohexadiene (6.3 mL, 10 eq., 67 mmol). The reaction mixture was stirred at room temperature for 2 h until complete conversion of the starting material. The crude mixture was filtered through a pad of Celite eluting with EtOAc and, after removal of the solvent under reduced pressure, the crude product was used in the next step without any further purification (1.83 g, 81% crude). UPLC-MS (method A): Rt. 1.77 min (TIC); ionization ES^+ 303 $[M+H]^+$.

^1H NMR (400 MHz, $CDCl_3$-*d*): δ 5.11–5.01 (m, 1H), 4.30–4.06 (m, 1H), 2.71 (t, *J* = 6.7 Hz, 2H), 1.86–1.74 (m, 1H), 1.71–1.56 (m, 13H), 1.55–1.37 (m, 12H).

Diacetone fructose triflate (6)

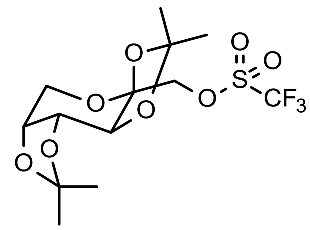

A solution of diacetone fructose (**5**) (5.0 g, 1.0 eq., 19.21 mmol) and pyridine (9.3 mL, 6.0 eq., 115.26 mmol) in DCM (100 mL) was treated with triflic anhydride (9.7 mL, 3.0 equiv, 57.63 mmol) at -20 °C. After stirring for 2 h at the same temperature, the reaction mixture was quenched with water, extracted with EtOAc, and the crude mixture was concentrated under reduced pressure. The residue was purified by flash chromatography eluting with cyclohexane/EtOAc (0 to 80%) to give the pure title compound as colourless oil (7.51 g, 99%). UPLC-MS (method A): Rt. 2.55 min (TIC); ionization ES^+ 393 $[M+H]^+$.

^1H NMR (400 MHz, $CDCl_3$-*d*): δ 4.66 (dd, *J* = 7.9, 2.7 Hz, 1H), 4.55 (d, *J* = 10.5 Hz, 1H), 4.43 (d, *J* = 10.5 Hz, 1H), 4.34 (d, *J* = 2.6 Hz, 1H), 4.27 (ddd, *J* = 7.8, 1.7, 0.8 Hz, 1H), 3.95 (dd, *J* = 13.0, 1.9 Hz, 1H), 3.81 (d, *J* = 13.0 Hz, 1H), 1.59 (s, 3H), 1.49 (s, 3H), 1.43 (s, 3H), 1.38 (s, 3H).

tert-Butyl 2-{[(tert-butoxy)carbonyl]amino}-6-[({4,4,11,11-tetramethyl-3,5,7,10,12-pentaoxatricyclo[7.3.0.0^2,^{6}]dodecan-6-yl}methyl)amino]hexanoate (7)

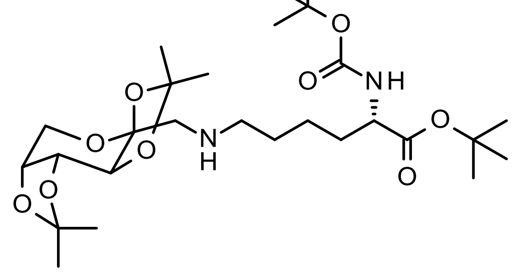

A solution of **4** (1.83 g, 1.0 eq., 6.05 mmol) and **6** (4.74 g, 2.0 eq., 12.1 mmol) in DMF (10 mL) was stirred at room temperature for 16 h until complete disappearance of the protected aminoacid **4**. The crude mixture was then treated with water and extracted with DCM (6×20 mL). After evaporation of the organics, the crude product was initially purified by flash chromatography eluting with DCM/MeOH (0 to 5%), and then by preparative HPLC to furnish the desired product as colourless oil (0.69 g, 21%).

UPLC-MS (method A): Rt. 2.34 min (TIC); ionization ES$^+$ 545 $[M+H]^+$.
^{1}H NMR (400 MHz, DMSO-d_6): δ 7.05 (d, *J* = 7.7 Hz, 1H), 4.55 (dd, *J* = 7.9, 2.5 Hz, 1H), 4.31 (d, *J* = 2.5 Hz, 1H), 4.22 (dd, *J* = 8.0, 1.5 Hz, 1H), 3.86 – 3.68 (m, 2H), 3.61 – 3.49 (m, 1H), 2.77 (d, *J* = 12.7 Hz, 1H), 2.67 – 2.54 (m, 3H), 2.49 – 2.40 (m, 2H), 1.65 – 1.49 (m, 3H), 1.48 – 1.24 (m, 32H).

Fructosyl lysine di-trifluoroacetate (8)

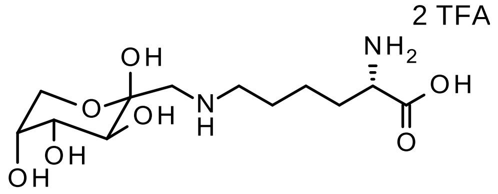

Compound **7** (0.21 g, 1.0 eq.) was stirred at room temperature in neat TFA (0.6 mL, 20 eq.) in the presence of catalytic amount of water (10%). The reaction was carefully monitored by UPLC-MS analysis. After 2 h upon complete conversion of the starting material, the reaction mixture was poured into cold Et_2O, thus obtaining a white precipitate. The white solid was suspended several times in Et_2O, the suspension sonicated, and then dried under vacuum to give the pure final compound as white solid (0.148 g, 90%). UPLC-MS (method A): Rt. 0.38 min (TIC); ionization ES$^+$ 309 $[M+H]^+$.
^{1}H NMR (600 MHz, D_2O): δ 3.98 (t, *J* = 6.3 Hz, 1H), 3.94 – 3.88 (m, 2H), 3.79 (dd, *J* = 9.8, 3.3 Hz, 1H), 3.66 (dd, *J* = 13.2, 2.2 Hz, 1H), 3.63 (d, *J* = 9.8 Hz, 1H), 3.23 – 3.16 (m, 2H), 3.08 – 3.01 (m, 2H), 1.99 – 1.84 (m, 2H), 1.76 – 1.65 (m, 2H), 1.52 – 1.37 (m, 2H).
^{13}C NMR (151 MHz, D_2O): δ 171.94, 162.87 (q, *J* = 35.8 Hz), 116.25 (q, *J* = 291.6 Hz), 95.34, 69.51, 69.21, 68.83, 63.86, 52.76, 52.60, 47.85, 29.18, 24.64, 21.42.
^{19}F NMR (565 MHz, D_2O): δ -74.62.

Acknowledgments
The authors thank Fabio Bertozzi for helpful discussions on the synthetic approaches, Sine Mandrup Bertozzi for preparative HPLC purifications, Luca Goldoni for recording ^{1}H-NMR spectra, and Marina Veronesi for recording ^{19}F-NMR spectra.
